# Differential expression of interferon-lambda receptor 1 splice variants determines the magnitude of the antiviral response induced by interferon-lambda 3 in human immune cells

**DOI:** 10.1371/journal.ppat.1008515

**Published:** 2020-04-30

**Authors:** Deanna M. Santer, Gillian E. S. Minty, Dominic P. Golec, Julia Lu, Julia May, Afshin Namdar, Juhi Shah, Shokrollah Elahi, David Proud, Michael Joyce, D. Lorne Tyrrell, Michael Houghton

**Affiliations:** 1 Li Ka Shing Institute of Virology and Department of Medical Microbiology and Immunology, University of Alberta, Edmonton, Alberta, Canada; 2 Alberta Diabetes Institute, University of Alberta, Edmonton, Alberta, Canada; 3 School of Dentistry, University of Alberta, Edmonton, Alberta, Canada; 4 Department of Oncology, University of Alberta, Edmonton, Alberta, Canada; 5 Department of Physiology and Pharmacology and Snyder Institute for Chronic Diseases, University of Calgary, Calgary, Alberta, Canada; The Ohio State University, UNITED STATES

## Abstract

Type III interferons (IFN-lambdas(λ)) are important cytokines that inhibit viruses and modulate immune responses by acting through a unique IFN-λR1/IL-10RB heterodimeric receptor. Until now, the primary antiviral function of IFN-λs has been proposed to be at anatomical barrier sites. Here, we examine the regulation of IFN-λR1 expression and measure the downstream effects of IFN-λ3 stimulation in primary human blood immune cells, compared with lung or liver epithelial cells. IFN-λ3 directly bound and upregulated IFN-stimulated gene (ISG) expression in freshly purified human B cells and CD8^+^ T cells, but not monocytes, neutrophils, natural killer cells, and CD4^+^ T cells. Despite similar *IFNLR1* transcript levels in B cells and lung epithelial cells, lung epithelial cells bound more IFN-λ3, which resulted in a 50-fold greater ISG induction when compared to B cells. The reduced response of B cells could be explained by higher expression of the soluble variant of IFN-λR1 (sIFN-λR1), which significantly reduced ISG induction when added with IFN-λ3 to peripheral blood mononuclear cells or liver epithelial cells. T-cell receptor stimulation potently, and specifically, upregulated membrane-bound *IFNLR1* expression in CD4^+^ T cells, leading to greater antiviral gene induction, and inhibition of human immunodeficiency virus type 1 infection. Collectively, our data demonstrate IFN-λ3 directly interacts with the human adaptive immune system, unlike what has been previously shown in published mouse models, and that type III IFNs could be potentially utilized to suppress both mucosal and blood-borne viral infections.

## Author summary

Type III IFNs (IFN-λs) are antiviral cytokines that are thought to act on specific subsets of cells, especially to protect mucosal barriers. Here, we demonstrate that IFN-λ3 differentially binds multiple human immune cell subsets, indicating the specific receptor subunit, IFN-λR1, is more broadly expressed in the human immune system, compared to published mouse models. IFN-λR1 expression increased after cellular activation, and antiviral responses were inhibited by a soluble version of the receptor. The direct interaction of IFN-λs with human immune cells, and specific regulation of IFN-λR1 expression, has broad mechanistic implications in the modulation of inflammatory or anti-cancer immune responses, and future antiviral therapies.

## Introduction

Type I and III interferons (IFNs) are induced in response to a variety of pathogens, and are responsible for the induction of a variety of ISGs that are essential for antiviral immune responses. While the type I IFN family was discovered in 1957 [1], the type III IFN family was discovered in 2003 [2–4]. There are four type III IFN (IFN-λ) family members: IFN-λ1 (IL-29), IFN-λ2 (IL-28A), IFN-λ3 (IL-28B) and IFN-λ4 [2, 3, 5, 6]. The majority of type III IFN studies have focused on the importance of type III IFNs in the defense against a number of viruses, especially at anatomical barriers [7–15]. In addition, multiple genome wide association studies have demonstrated the importance of the *IFNL3/4* locus in both IFN-α treatment response and the natural clearance of the hepatitis C virus (HCV) [16–18]. Type III IFNs can also significantly dampen inflammation in mouse models of allergic asthma, colitis, and autoimmune arthritis [19–22]. Differences in biological activities between type I and type III IFNs likely relate to differences in cell-type specific receptor expression, and potency and kinetics of signaling, where IFN-λs induce a slower, prolonged, lower magnitude response [23, 24]. It has been proposed that IFN-λs could act as an initial defense to inhibit virus replication without causing inflammation, before type I IFNs are induced [25, 26].

All type III IFN family members signal through a unique heterodimeric receptor comprised of IFN-λR1 (IL-28RA) and IL-10RB [2, 3, 27]. Similar to type I IFNs, type III IFNs induce ISGs by activating JAK1 and TYK2, which associate with IFN-λR1 and IL-10RB, respectively, leading to the phosphorylation of STAT1/STAT2 and ISG induction [28–30]. Unlike the type I IFN receptor (IFNAR1/2) and IL-10RB, which are ubiquitously expressed on virtually all nucleated cells, IFN-λR1 expression is more restricted. *IFNLR1* transcripts and/or IFN-λ responsiveness has been observed in epithelial cells of the lung, liver, and gut [28, 31–33], endothelial cells of the blood brain barrier [11] and trophoblasts within a placenta [34]. Multiple splice variants of *IFNLR1* have been described in human cells [3, 4, 35], but the majority of work has focused on the full length, membrane form (*mIFNLR1*), with little data reported on the biological effects of the soluble form (*sIFNLR1*) in which the transmembrane domain is deleted. Within the immune system, human plasmacytoid dendritic cells (pDCs) strongly respond to IFN-λs, but little or no IFN-λR1 transcript has been found in monocytes, natural killer cells, or T cells [7, 35–44]. Transcripts for *IFNLR1* are detectable in human, but not mouse B cells [20, 45], but the human B cell response to IFN-λs has not been consistently demonstrated [7, 35, 44, 45]. In mouse models of infection or autoimmunity, neutrophils are the major immune cell type that express high levels of *Ifnlr1* transcripts and can potently respond to IFN-λs [19, 20, 25, 46], but more work is needed to determine if IFN-λs directly stimulate ISGs in human neutrophils.

Previously, we demonstrated that IFN-λ3 inhibited human B cell antibody production and decreased the Th2 response to an H1N1 influenza vaccine antigen [47], but it is not clear which human immune cells directly respond to IFN-λ3. Here, we investigate and quantify expression of IFN-λR on human immune cells and correlate these findings with ISGs induced by IFN-λ3. We demonstrate that human adaptive immune cells express both IFN-λR1 variants (mIFN-λR1, sIFN-λR1), where sIFN-λR1 inhibits ISG induction by IFN-λ3. In addition, we show mIFN-λR1 expression varies between cell subsets and can be upregulated by activation of immune cell receptors including the T-cell receptor (TCR), B-cell receptor (BCR) and Toll-like receptors (TLR). In purified activated CD4^+^ T cells, IFN-λ3 pretreatment leads to antiviral ISG induction and a significant decrease in HIV-1 infection. Taken together, these results show that unlike in mice, IFN-λ3 directly regulates the human adaptive immune system and may be exploited in the future to promote type 1 and antiviral responses and dampen type 2 immune responses.

## Results

### IFN-λR1 is differentially expressed among peripheral human immune cell subsets

**Fig 1:**
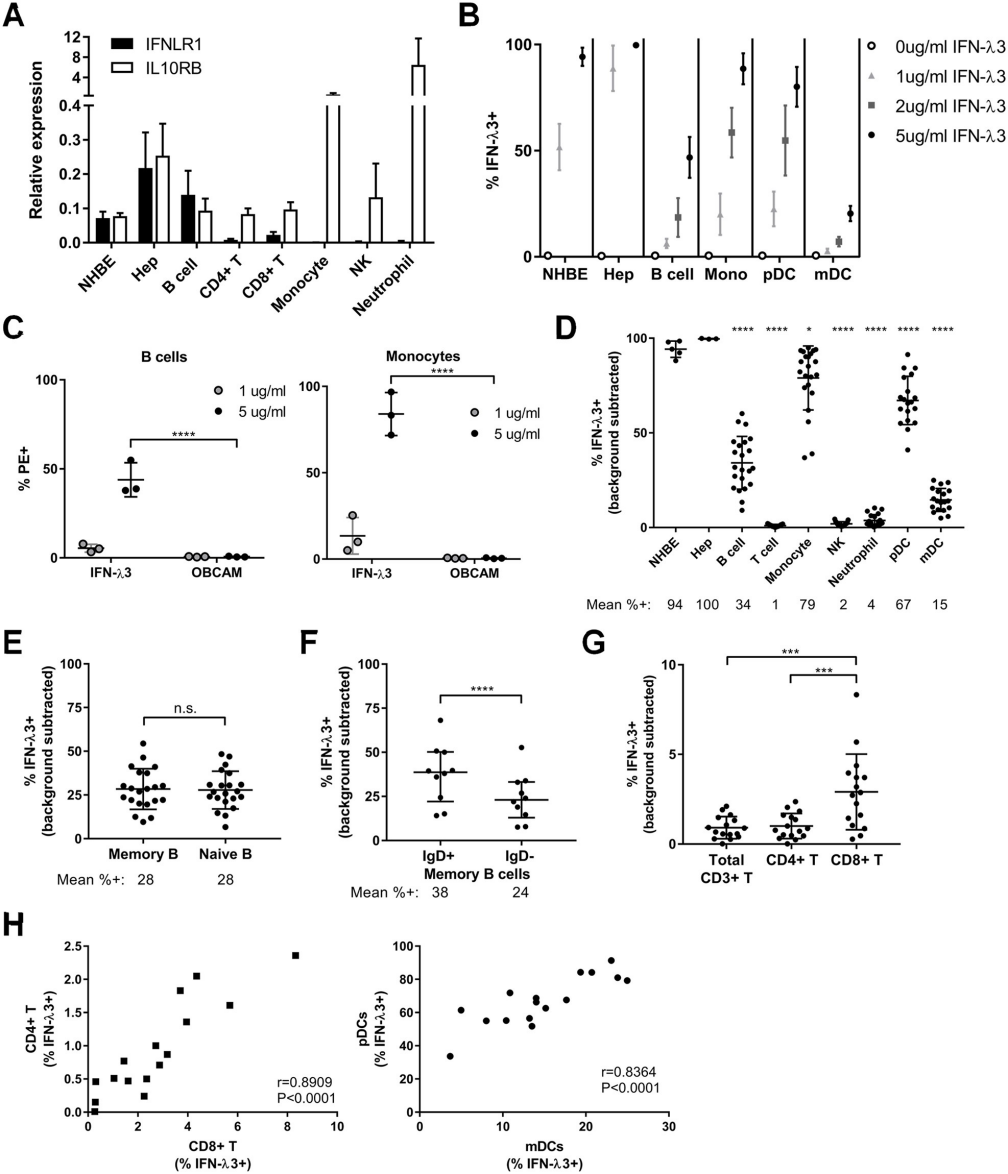
Differential IFN-λR expression and IFN-λ3 binding among primary human immune and epithelial cells. A) Normalized expression values for each subunit of the IFN-λR as determined by RT-qPCR for normal human bronchial epithelial cells (NHBE), primary hepatocytes (hep) or immune cells purified from healthy human donor blood. Graph shows mean + SEM from 3–7 different donors for each population. Results were normalized to the geomean of HPRT1 and RPL13A reference genes. B-H) IFN-λ3 binding was quantified via flow cytometry as described in the Materials and Methods. B) Dose curves of adding 1, 2 or 5 μg/ml IFN-λ3 to epithelial cells or total human PBMCs. 0 μg/ml IFN-λ3 refers to adding the secondary antibody alone. Graph shows mean +/- SD for 3–7 different donors. C) Binding percentages as detected by flow cytometry for IFN-λ3 or a similarly His-tagged control protein (OBCAM) where means +/- SD are shown. D-H) Quantified IFN-λ3 (5 μg/ml) binding percentages to each cell type where each dot represents a different healthy individual and background binding of the secondary antibody alone was subtracted. In D), statistical significance results were identical when comparing either NHBE or hep to all other immune cell subsets. All comparisons are shown in S1 Table. H) Pearson correlation coefficients (r) calculated when comparing IFN-λ3 binding to different immune cell subsets. All comparisons are shown in S2 Table. *, P<0.05, ***, P<0.001, ****, P<0.0001, two-way (C) or one-way (D, G) ANOVA with Tukey’s multiple comparisons test, paired t-test (E-F).

Recent type III IFN studies have focused on their antiviral or protective effects in epithelial cells at barrier sites, but consensus is lacking regarding which human immune cells express the IFN-λR. We first quantified transcript levels of both subunits of IFN-λR (*IFNLR1* and *IL10RB*) in highly pure immune cell subsets from blood of healthy individuals, primary human hepatocytes, and normal human bronchial epithelial cells (NHBE). We found, as expected, ubiquitous *IL10RB* expression, with the highest levels found in monocytes and neutrophils (Fig 1A). The highest expression of *IFNLR1* transcripts were found in epithelial cell types and B cells, while CD4^+^ and CD8^+^ T cells expressed less *IFNLR1* mRNA, and monocytes, natural killer cells, and neutrophils had little or barely detectable expression levels of *IFNLR1* transcript (Fig 1A). CD8^+^ T cells had significantly greater expression of *IFNLR1* compared to CD4^+^ T cells (P = 0.0021). This is the first report of differential expression of *IFNLR1* in human CD4^+^ versus CD8^+^ T cells. Our results contrast those in mice where *Ifnlr1* transcript expression is highest in neutrophils and little or no expression can be detected in all other immune cell types [20, 25].

### IFN-λ3 binds to both epithelial cells and specific immune cell subsets

To date, studies on IFN-λR1 biology have been limited due to a lack of sensitive reagents to measure receptor protein expression. We recently developed a flow cytometry binding assay [48], which measures IFN-λ3 binding to the cell surface as a surrogate of IFN-λR1 expression. We quantified the binding of 6 His-tagged IFN-λ3 to immune cell subsets within peripheral blood, as well as primary liver and lung epithelial cells. Our flow cytometry gating strategy for PBMCs and neutrophils is shown in S1 and S2 Figs. Multiple immune cell subsets bound IFN-λ3 in a dose-dependent manner, and the maximum binding percentage was dependent on the cell type (Fig 1B). Almost 100% of hepatocytes and NHBE cells bound IFN-λ3 at the highest dose tested (Fig 1B), and the amount of IFN-λ3 bound (median PE value) was 4.4–11.7 fold greater than what bound to immune cell subsets (S3A Fig). The binding results were very reproducible within a single donor; the percentage of monocytes or B cells that bound IFN-λ3 did not vary substantially when the same assay was repeated at least six months later (S3B Fig). An unrelated 6 His-tagged protein (OBCAM) did not significantly bind any of the cell types examined (Fig 1C and S3C Fig). IFN-λ3 also did not significantly bind Huh7 *IFNLR1* knockout cells, which were previously shown to be unresponsive to IFN-λ3 stimulation [49] (S3D Fig). Consistent with previous reports that pDCs respond to IFN-λ stimulation [41, 43, 44], IFN-λ3 bound to pDCs at high levels (Fig 1B and 1D). B cells, monocytes, pDCs, and mDCs all bound IFN-λ3, whereas little IFN-λ3 bound to total T cells, NK cells, and neutrophils (Fig 1B and 1D). IFN-λ3 bound a significantly higher percentage of hepatocytes and NHBE cells compared with all immune cell subsets tested (Fig 1D, S1 Table). While there was no difference in IFN-λ3 binding between memory and naïve B cells, we observed significantly higher binding of IFN-λ3 to IgD^+^ versus IgD^-^ memory B cells (Fig 1E and 1F). CD8^+^ T cells also bound significantly more IFN-λ3 than CD4^+^ T cells (Fig 1G), consistent with the greater *IFNLR1* transcripts measured in CD8^+^ T cells (Fig 1A). Next, we examined whether the percentage of IFN-λ3 binding correlated between immune cell subsets within each donor. We found there was significant correlation between cell types, especially within the same immune cell lineage. For example, the levels of IFN-λ3 binding to CD4^+^ T cells positively correlated with the percentage bound to CD8^+^ T cells, and similarly, the binding of IFN-λ3 to pDCs positively correlated with the binding to mDCs (Fig 1H). Correlation results between immune cell subsets are shown in S2 Table and S4 Fig. The binding of IFN-λ3 to the cell surface matched the relative expression of *IFNLR1* transcripts quantified in Fig 1A for most immune cell subsets. The exception was monocytes where little *IFNLR1* transcript was detectable, but IFN-λ3 binding was observed. Interestingly, despite comparable *IFNLR1* mRNA levels in B cells and NHBE cells (Fig 1A), significantly higher amounts of IFN-λ3 bound to NHBE cells than B cells (P<0.0001) (Fig 1B and 1D and S3A Fig). Collectively, these results show that primary epithelial cells and specific human immune cell subsets bind IFN-λ3, but epithelial cells bind IFN-λ3 at a much higher level.

### IFN-λ3 binding leads to ISG induction in both epithelial cells and specific immune cell subsets

**Fig 2:**
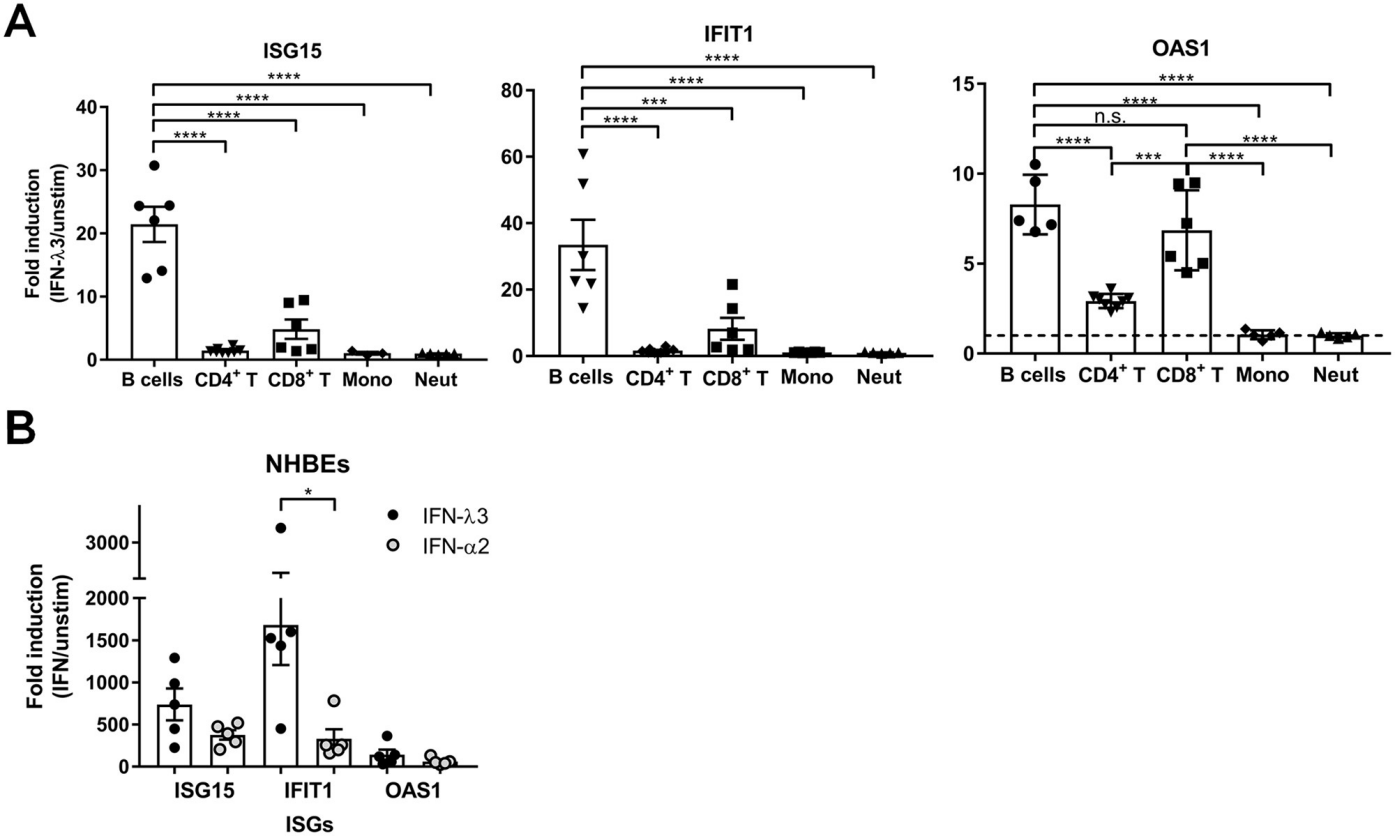
IFN-λ3 mediated ISG induction in primary human lung epithelial cells, B cells and T cells, but not monocytes or neutrophils. A-B) RT-qPCR quantification of ISG15, IFIT1, and OAS1 induced by IFN-λ3 (100 ng/ml) purified human immune cell subsets (A) or normal human bronchial epithelial cells (NHBEs) (B). All cell types were cultured with or without IFN-λ3 for 24 hrs, except neutrophils (5 hrs). Graphs show fold induction relative to unstimulated cells after normalization to the geomean of HPRT1 and RPL13A reference genes. Bars represent mean +/- SEM from 3–8 different donors. n.s., not significant, *, P<0.05. ***, P<0.001, ****, P<0.0001, one-way ANOVA, Tukey’s multiple comparisons test (A), paired t-test (B).

Previous studies that examined IFN-λR expression and IFN-λ stimulation of human immune cells had conflicting results [35, 39, 44, 45, 50]. Since we observed dramatic differences in IFN-λ3 binding by immune cell subsets, we next determined whether the amount of IFN-λ3 bound by each subset mirrored the relative induction of ISGs upon IFN-λ3 stimulation. We compared IFN-λ3 responses in highly pure B cells, monocytes, CD4^+^ T cells, CD8^+^ T cells, or neutrophils freshly isolated from peripheral blood of healthy donors. Representative results of the purity of our isolated cell subsets are shown in S2 and S5 Figs. For all cell types except neutrophils, we added recombinant IFN-λ3 overnight to induce ISG expression. Neutrophils were treated with IFN-λ3 for 5 hours since the majority of neutrophils die during overnight culture *in vitro* [51–53]. Among immune cells, the greatest ISG response to IFN-λ3 was seen in B cells, consistent with their expression of *IFNLR1* and IFN-λ3 binding potential (Fig 2A). Low levels of ISGs were induced by IFN-λ3 in CD4^+^ T cells with the highest induction of *OAS1* (mean 2.9 fold upregulation in IFN-λ3 treated versus unstimulated control), whereas all ISGs tested were induced by IFN-λ3 in CD8^+^ T cells (mean 4.8 to 8.2 fold upregulation in IFN-λ3 treated versus unstimulated control). Baseline expression of all 3 ISGs was not statistically different between B cells, CD4^+^ T cells and CD8^+^ T cells, therefore the higher response in B cells was not due to lower ISG expression prior to IFN-λ3 stimulation (S6A Fig). In contrast, neutrophils had significantly greater baseline IFIT1 and ISG15 expression compared to B and T cells, in agreement with published microarray and single cell RNA sequencing data [54, 55] (S6A Fig). OAS1 expression was not significantly different between B cells and neutrophils or monocytes. Overall, our results indicate both B and T cells can directly respond to IFN-λ3, but with different magnitudes. Both monocytes and neutrophils failed to upregulate ISGs in response to IFN-λ3 (Fig 2A), demonstrating that the IFN-λ3 we detected bound to monocytes in our binding assay does not induce ISG induction, at least under the conditions tested. All immune cell types tested responded to our positive control IFN-α2 (S6B Fig).

**Fig 3:**
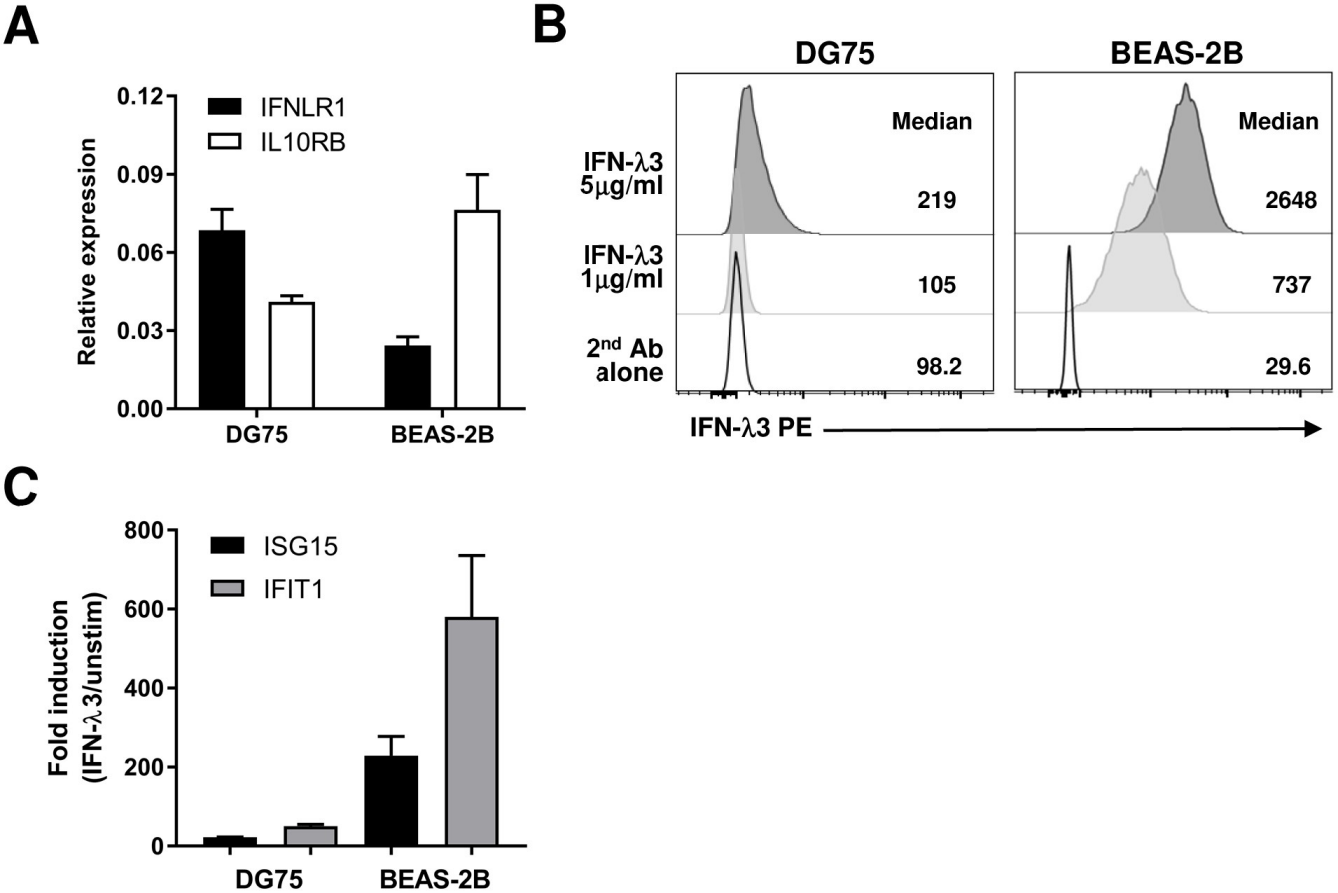
Greater IFN-λ3 cell surface binding and ISG induction in BEAS-2B bronchial epithelial cell line compared to DG75 B cell line mimics results seen in primary cells. A) Normalized expression values for each subunit of the IFN-λR as determined by RT-qPCR for DG75 or BEAS-2B cell lines. Results were normalized to the geomean of *HPRT1* and *RPL13A* reference genes. B) IFN-λ3 flow cytometry binding assay results for 0, 1, or 5 μg/ml His-tagged IFN-λ3 added to DG75 or BEAS-2B cells. C) RT-qPCR quantification of *ISG15* and *IFIT1* mRNA in DG75 or BEAS-2B cells induced by IFN-λ3 (100 ng/ml) as compared to unstimulated cells after 24 hrs incubation. Bar graphs (A, C) show means + SEM from 2 independent experiments and flow cytometry histograms (B) are representative of 2 independent binding assays.

Since we observed greater IFN-λ3 binding to NHBE cells compared to B cells despite higher relative *IFNLR1* transcript expression in B cells, we next quantified ISG induction by IFN-λ3 in NHBE cells. When NHBE cells were treated with IFN-λ3 overnight, high levels of ISGs were induced (Fig 2B). At this time point, ISG induction by IFN-λ3 was significantly higher than the levels we observed with IFN-α2 treatment for most genes tested (Fig 2B). There was up to 50-fold higher ISG induction by IFN-λ3 in NHBE cells compared to B cells (Fig 2A and 2B). We confirmed NHBE and B cells had comparable baseline ISG expression (S6A Fig). This result matched our cell surface IFN-λ3 binding quantification, which was ~5 fold higher in NHBE cells compared to B cells (S3A Fig). We then recapitulated this phenomenon in cell lines. The DG75 B cell line expressed on average 2.8 fold greater *IFNLR1* mRNA than the BEAS-2B bronchial lung epithelial cell line, although greater *IL10RB* expression was found in BEAS-2B cells (Fig 3A). Analogous to our results in primary cells, BEAS-2B lung epithelial cells bound dramatically more IFN-λ3 at the cell surface compared to DG75 B cells with 7–12 fold higher median fluorescent intensities detected (Fig 3B). This increased IFN-λ3 cell surface binding translated to 10–11 fold greater ISG induction in BEAS-2B cells compared to DG75 B cells in response to overnight IFN-λ3 treatment (Fig 3C). Our results demonstrated that our IFN-λ3 binding assay is a useful tool to predict IFN-λ3 responsiveness. While previous studies have solely quantified *IFNLR1* mRNA and because a sensitive antibody is not commercially available, one must be cautious that total *IFNLR1* transcript levels do not necessarily correlate with IFN-λ3 induced ISGs.

### Soluble IFN-λR1 directly binds cells to increase IFN-λ3 binding to the surface but inhibits ISG induction

**Fig 4:**
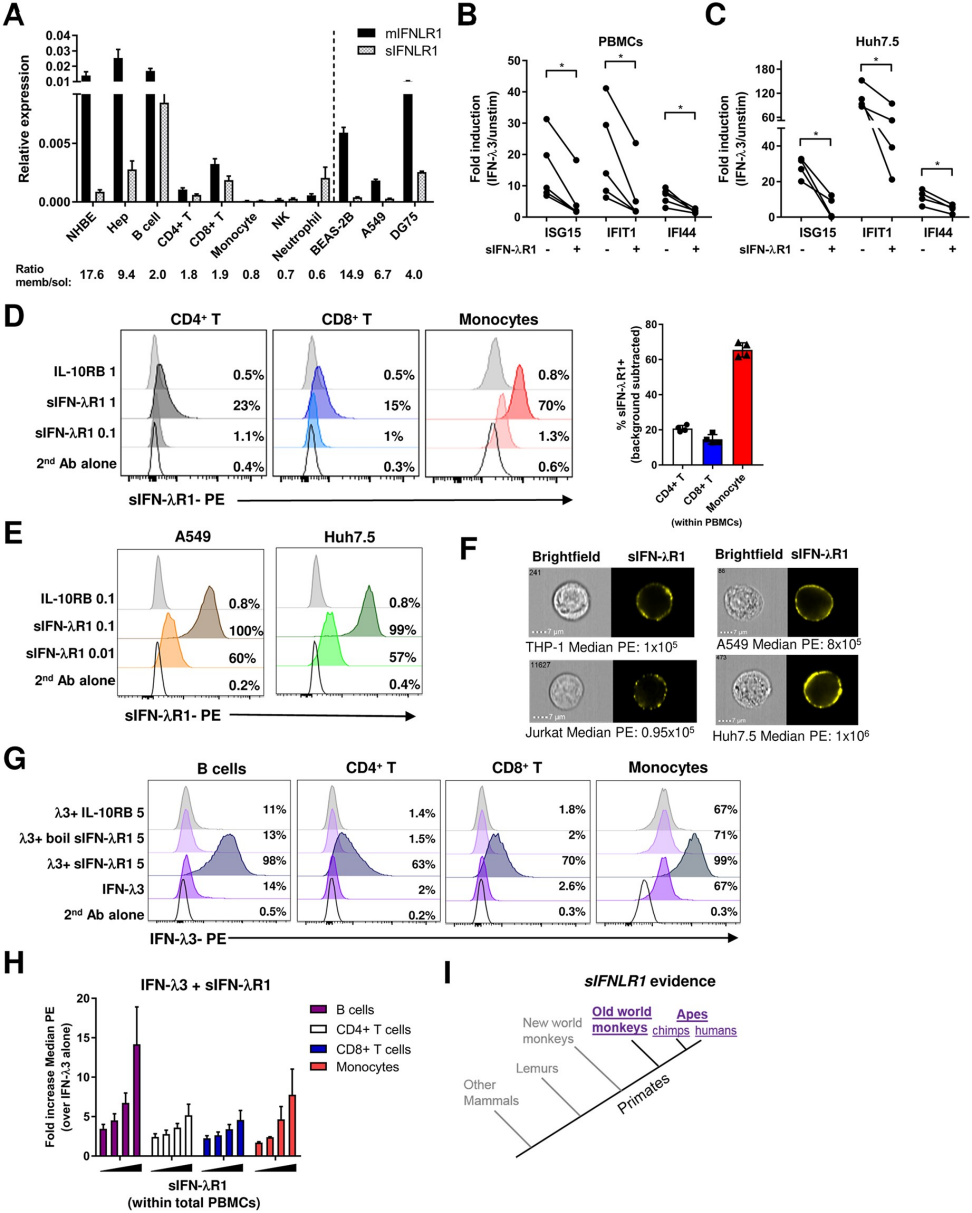
Soluble IFN-λR1 found in primates binds to the cell surface to increase binding of IFN-λ3 but inhibits ISG induction. A) Normalized expression values for each variant of *IFNLR1* (membrane, full length (*mIFNLR1*), or small, soluble (*sIFNLR1*)) as determined by RT-qPCR for normal human bronchial epithelial cells (NHBE), hepatocytes (hep) or immune cells purified from healthy human donor blood, or cell lines (BEAS-2B or A549 lung epithelial or DG75 B cell). Means + SEM are shown from 3–6 different donors (primary cells) or 2–3 independent experiments (cell lines). Results were normalized to the geomean of *HPRT1* and *RPL13A* reference genes. B-C) Fold induction of 3 ISGs (ISG15, IFIT1, IFI44) by IFN-λ3 (10 ng/ml) treatment of total PBMCs (B) or Huh7.5 hepatocytes (C) relative to unstimulated cells with or without simultaneous addition of recombinant sIFN-λR1 (100 ng/ml (PBMC), 1000 ng/ml (Huh7.5)). Each dot represents a different individual or experiment. D-E) Quantification of binding of recombinant sIFN-λR1 or control IL-10RB to individual cell subsets within total PBMCs (D), or to A549 or Huh7.5 cell lines (E). Binding was detected by anti-Fc PE antibody. Histograms are representative of results from 4 different PBMC donors (bar graph right panel D), or from 2–3 independent experiments (E). F) Imaging flow cytometry visualization of binding of sIFN-λR1 (500 ng/ml (A549, Huh7.5), 1 μg/ml (THP-1, Jurkat)) to 4 cell lines. The median PE intensity is shown below each representative image from 6,000–12,000 total cells acquired from 2–3 independent experiments. G-H) IFN-λ3 binding to cell subsets within PBMCs where IFN-λ3 (2 μg/ml) was added with or without sIFN-λR1 (0.5–5 μg/ml +/- boiling) or IL-10RB (5 μg/ml). Percent IFN-λ3 bound and fold increase in median PE fluorescence are representative from 2–4 donors (G) or plotted as means + SD from 2–4 different donors (H). I) The presence of a soluble *IFNLR1* variant within primates and lower mammals. Gray indicates no annotation and no transcript detectable in deposited RNA sequencing data, and purple indicates both annotation and detection of RNA transcript in multiple cell types from deposited RNA sequencing data. *, P<0.05, paired t-tests (B-C).

To further examine the discrepancy between high and similar levels of *IFNLR1* transcript seen in both B cells and lung epithelial cells, but low B cell ISG expression in response to IFN-λ3, we determined if sIFN-λR1 plays a role in controlling ISG responses. This variant lacks a transmembrane domain and was detectable in cell line supernatants when overexpressed [35]. Little is known about the regulatory role of sIFN-λR1 in IFN-λ biology except that pre-incubating 100–1000 fold excess sIFN-λR1 with IFN-λ1 inhibited MHC class I upregulation on the HepG2 hepatocyte cell line [35]. We used our previously designed RT-qPCR assay to specifically quantify either the full length membrane form (*mIFNLR1*) or *sIFNLR1* [48]. Using this assay, we found all immune cells tested had higher levels of *sIFNLR1* relative to *mIFNLR1*, when compared to epithelial cells. The membrane/soluble receptor transcript ratio was 0.6–2 in immune cells, whereas in lung or liver epithelial cells it was 9.4–17.6 (Fig 4A). B cells had similar *mIFNLR1* mRNA expression, but higher *sIFNLR1* levels compared to epithelial cells. CD8^+^ T cells expressed the next highest transcript level of *mIFNLR1* at 3-fold higher levels than CD4^+^ T cells. Monocytes and NK cells expressed very little or no *IFNLR1* mRNA of either variant. Neutrophils were the only cell type tested where there was higher *sIFNLR1* than *mIFNLR1* expression (Fig 4A). In agreement with our data with primary T and B cells, we had previously found Jurkat T cell and Raji B cell lines also had low membrane/soluble *IFNLR1* ratios of ~2–3 [48]. We extended our cell line results to demonstrate that the lung BEAS-2B and A549 epithelial cell lines had a much higher membrane/soluble *IFNLR1* transcript ratio of 14.9 and 6.7 respectively, while DG75 B cells had a lower ratio of 4.0 (Fig 4A). Our results indicate that the varied expression of *sIFNLR1* may relate to extent of the ISG response induced by IFN-λ3.

To examine if sIFN-λR1 regulates IFN-λ3-mediated ISG responses, we added recombinant sIFN-λR1 protein alongside IFN-λ3 to PBMCs and then quantified ISG induction. The addition of sIFN-λR1 dramatically inhibited ISG induction by an average of 54–78% (Fig 4B). This inhibition of IFN-λ3 activity was not unique to immune cells because sIFN-λR1 similarly inhibited IFN-λ3-mediated ISG induction in the Huh7.5 hepatocyte cell line (Fig 4C). Since soluble cytokine receptors can act to prevent cytokine binding to the cell surface, or can act directly at the cell surface interacting with co-receptors [56], we first determined if sIFN-λR1 could bind the cell surface in the absence or presence of IFN-λ3 cytokine. Surprisingly, sIFN-λR1 bound both primary immune cells within PBMCs (Fig 4D), and epithelial cell lines (Fig 4E) in the absence of IFN-λ3. This binding was not due to the Fc tag on recombinant IFN-λR1, since excess human IgG was added first to block Fc receptors, and recombinant IL-10RB with the same Fc tag did not bind any cell type tested (Fig 4D and 4E). Among peripheral immune cells, more primary monocytes bound sIFN-λR1 than any other cell type, but A549 and Huh7.5 epithelial cell lines required ~100-fold less sIFN-λR1 protein to bind similar levels of sIFN-λR1 as monocytes (0.01 μg/ml epithelial cells versus 1 μg/ml for monocytes) (Fig 4D and 4E). Due to the secondary anti-Fc antibody binding to surface IgG on primary B cells, we did not include B cell results in our analysis. We visualized sIFN-λR1 binding to the cell surface of four cell lines using imaging flow cytometry. We observed a distinct cell surface staining pattern with higher levels of sIFN-λR1 on the surface of A549 and Huh7.5 epithelial cell lines compared to THP-1 monocytes and Jurkat T cells (median 8–10 fold greater despite 2-fold less sIFN-λR1 added) (Fig 4F). Addition of IFN-λ3 protein to cells simultaneously with sIFN-λR1 did not increase sIFN-λR1 binding to Huh7.5 cells (S7A Fig). Taken together, we have shown for the first time that sIFN-λR1 can bind multiple cell types without requiring previous interaction with IFN-λ cytokine.

After observing striking differences in binding of sIFN-λR1 to various cell types, we next utilized our IFN-λ3 binding assay to determine if sIFN-λR1 binding to the cell surface affects IFN-λ3 binding. The addition of recombinant sIFN-λR1 with IFN-λ3 to PBMCs led to 5–15 fold greater binding of IFN-λ3 to the surface of B cells, monocytes, and T cells compared to IFN-λ3 alone at the highest dose tested (Fig 4G and 4H). Addition of sIFN-λR1 also increased binding of IFN-λ3 to Huh7.5 cells in a dose-dependent manner (S7B Fig). Allowing sIFN-λR1 to bind first to cells before adding IFN-λ3, or adding them simultaneously, resulted in the equivalent increased binding of IFN-λ3 compared to adding cytokine alone (S7C Fig). Recombinant IL-10RB or denatured sIFN-λR1 at the same concentrations did not increase IFN-λ3 binding to any cell type tested (Fig 4G). These results could help explain why B cells are less responsive to IFN-λ3 compared to lung epithelial cells despite similar *IFNLR1* transcript expression. Taken together, our data shows sIFN-λR1 increased IFN-λ3 binding to the cell surface, potentially through interactions with IL-10RB, the second subunit of the IFN-λR. This binding would prevent ISG induction though, because the cytoplasmic tail, not present in sIFN-λR1, is required for downstream signaling [29]. Lower levels of sIFN-λR1 at epithelial barriers could explain why those cell types are especially responsive to type III IFNs.

### Soluble *IFNLR1* evolved late in evolution with detectable transcripts in specific primates

Our data describing which human immune cell express total *IFNLR1* transcripts contrast published mouse cell *Ifnlr1* expression, but previous studies had only measured *sIFNLR1* transcripts in human cells [3, 4, 35]. We next investigated whether other primates and lower mammals such as mice also encoded for a soluble form of *IFNLR1*. We found that the *sIFNLR1* transcript variant missing the transmembrane domain (exon 6) is only annotated in primates, specifically most old world monkeys and all apes, but not in new world monkeys, lemurs, tarsiers and lower mammals with the exception of the guinea pig (Fig 4I and S3 Table). To examine if annotations were missing for *sIFNLR1* and to find experimental evidence of *sIFNLR1* expression, we BLAST searched the NCBI Sequence Read Archive (SRA) using a 120 nucleotide sequence spanning the end of exon 5 and beginning of exon 7 for each species of interest. We found detectable *sIFNLR1* transcripts in experiments with cells from apes and certain old world monkeys in multiple tissues including epithelial cells and immune cells, but *sIFNLR1* transcripts could not be detected in any experimental data deposited for lower mammals, including the potential variant annotated in guinea pigs. A full summary of species studied and example data accession numbers are listed in S3 Table. These results are consistent with the late evolution of the soluble variant of IFN-λR1 in mammals, with only the closest relatives of humans having experimental evidence for the expression of *sIFNLR1*.

### Stimulation of B cells and neutrophils upregulates IFN-λR1 expression

**Fig 5:**
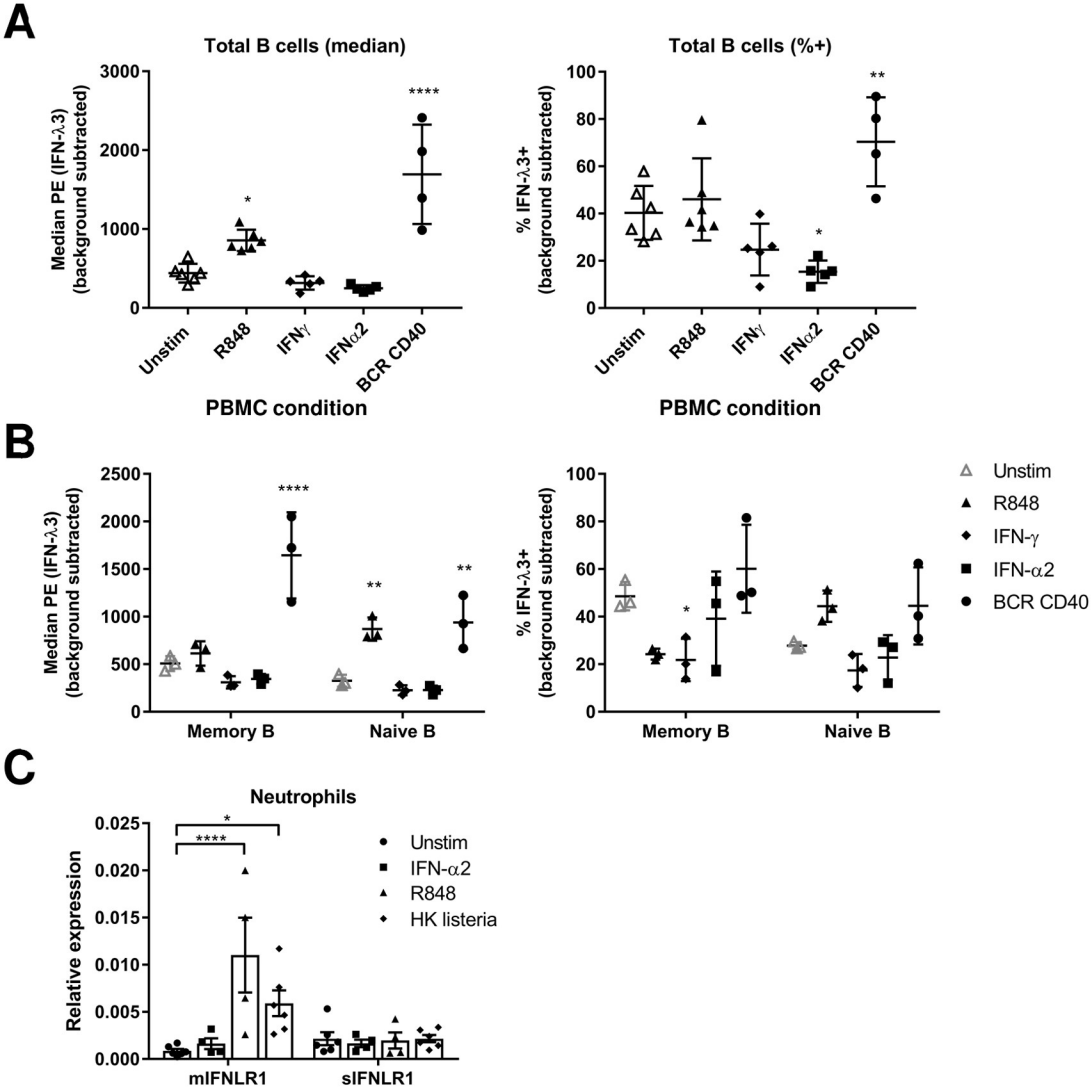
BCR or TLR activation upregulate IFN-λ3 binding and IFN-λR1 expression. A-C) IFN-λ3 (5 μg/ml) binding to LIVE/DEAD^-^ CD20^+^ B cells (A-B, memory (CD27^+^), naïve (CD27^-^)) or CD3^+^ CD4^+^ or CD8^+^ T cells (C) within total PBMCs that had been cultured for 3 days either unstimulated (unstim), or with the following stimuli: R848 (1 μg/ml), IFN-γ (10 ng/ml), IFN-α2 (1000 IU/ml) or anti-IgM/IgG/IgA (BCR, 10 μg/ml) and anti-CD40 (5 μg/ml). The percent IFN-λ3^+^ or median PE fluorescence is shown after background subtraction of secondary antibody alone with means +/- SD. C) RT-qPCR quantification of *sIFNLR1* and *mIFNLR1* transcripts in purified neutrophils that were left unstimulated (unstim) or stimulated with IFN-α2 (1000 IU/ml), R848 (1 μg/ml) or heat killed listeria (HK listeria) supernatant (1:100) for 5 hrs. *, P<0.05, **, P<0.01, ****, P<0.0001, one-way ANOVA (A) or two-way ANOVA (B-C), Dunnett’s multiple comparisons test between each treatment and unstimulated. Only significant comparisons are noted and each symbol represents a different individual donor.

The differential expression of IFN-λR1 on the surface of different human immune cell subsets implied that IFN-λR1 expression can be regulated. We therefore examined whether IFN-λR1 expression could be altered by adding various stimuli to B cells or neutrophils. B cells were chosen because they represent cells that express high levels of *IFNLR1*, while neutrophils have little *IFNLR1* transcript expression. Previous work had shown that IFN-α significantly upregulated *IFNLR1* mRNA in human hepatocytes [9], so we tested whether this regulation also occurred in immune cells. Stimulation of PBMCs for 3 days with anti-BCR (IgM/IgG/IgA) and anti-CD40 or the TLR7/8 ligand R848, but not the cytokines IFN-γ or IFN-α2, increased IFN-λ3 binding to total B cells (Fig 5A). Surprisingly, IFN-α2 treatment significantly decreased the percent of total B cells binding IFN-λ3 by 62% on average (Fig 5A). We performed a similar analysis to compare naïve and memory B cells within PBMCs. R848 treatment significantly upregulated IFN-λ3 binding fluorescence to naïve B cells, but not memory B cells, whereas IFN-γ addition specifically decreased IFN-λ3 binding to memory B cells (Fig 5B).

Similar to B cells, when purified human neutrophils were stimulated with R848 for 5 hours, the transcript for *mIFNLR1* was significantly upregulated (Fig 5C). The addition of heat killed listeria bacteria supernatant, which would stimulate multiple pattern recognition receptors, also significantly upregulated *mIFNLR1* transcript expression (Fig 5C). *sIFNLR1* transcript expression was not significantly altered in any neutrophil stimulation assay. Collectively, our data demonstrate that receptor stimulation of B cells and neutrophils can upregulate surface expression of IFN-λR1. Therefore, during an infection or inflammatory state, multiple immune cell types could have greater responsiveness to type III IFNs.

### Stimulation of CD4^+^ T cells via the TCR upregulates IFN-λR1 expression and IFN-λ3 induced ISG expression

**Fig 6:**
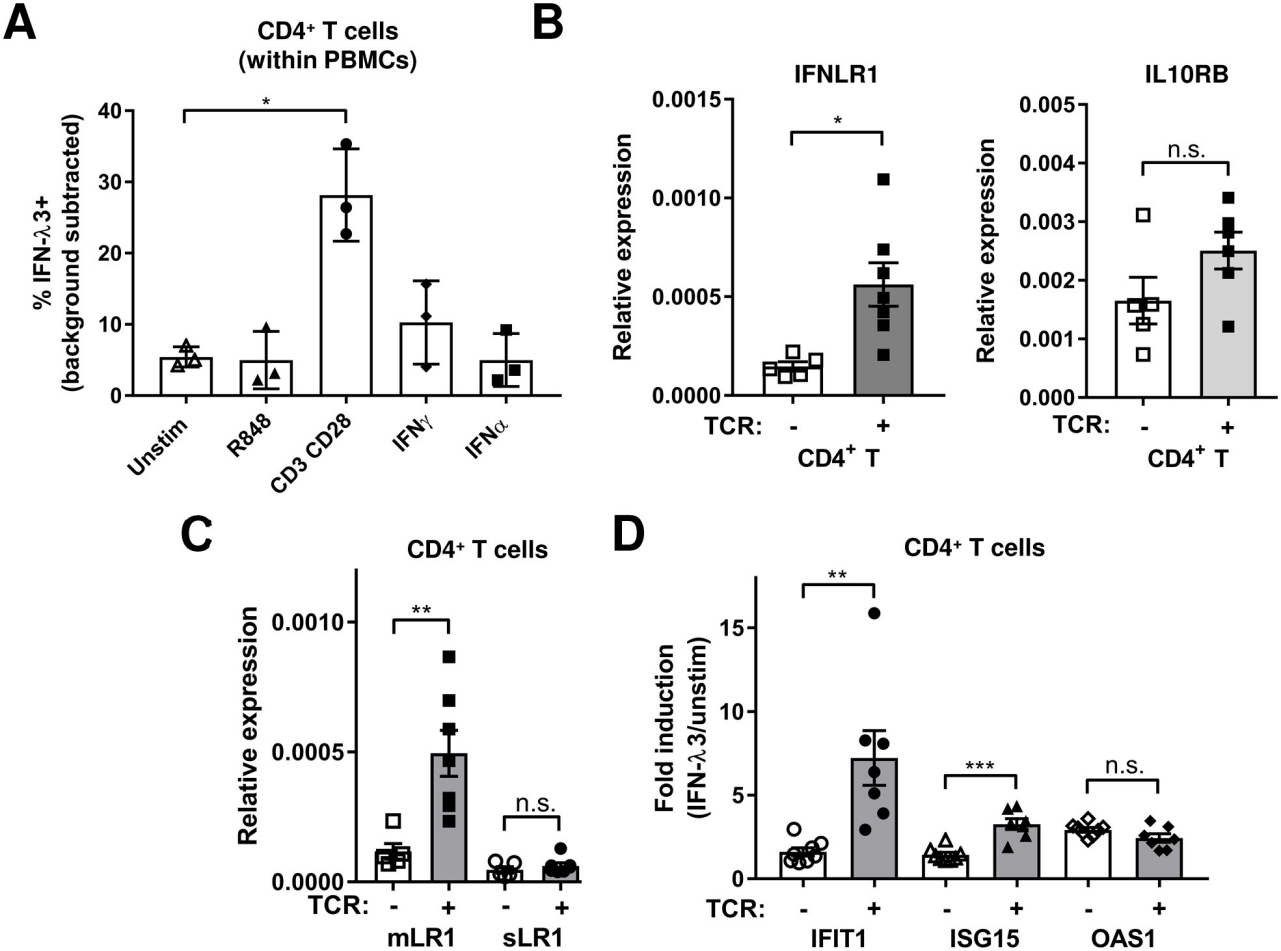
TCR stimulation upregulates IFN-λ3 binding and IFN-λR1 expression leading to greater ISG induction in CD4^+^ T cells. A) IFN-λ3 (5 μg/ml) binding to LIVE/DEAD^-^ CD4^+^ T cells within total PBMCs that had been cultured for 3 days in either media only (unstimulated (unstim)), or with the following stimuli: R848 (1 μg/ml), anti-CD3 (plate bound 1.5 μg/ml) and soluble anti-CD28 (1 μg/ml), IFN-γ (10 ng/ml) or IFN-α2 (1000 IU/ml). B-C) Total *IFNLR1*, *IL10RB* (B), full length membrane (*mLR1*) or soluble (*sLR1*) *IFNLR1* variant (C) transcript expression in CD4^+^ T cells sorted after 3 days of PBMC culture with or without anti-CD3/anti-CD28 stimulation (TCR). D) ISG induction in sorted CD4^+^ T cells treated with IFN-λ3 (100 ng/ml) for 24 hrs after sorting from PBMCs which had previously been cultured in unstimulated or anti-CD3/anti-CD28 stimulated (TCR) conditions for 3 days. Fold induction of 3 ISGs are shown. Graphs show means +/- SD (A) or SEM (B-D) with each symbol representing a different healthy individual. All RT-qPCR results are normalized to *B2M* reference gene. n.s., not significant, *, P<0.05, **, P<0.01, ***, P<0.001, one-way ANOVA, Dunnett’s multiple comparison test comparing to unstimulated (A), unpaired t-test (B-D).

Given that BCR and TLR activation upregulated IFN-λR1 expression on B cells and neutrophils, we hypothesized that the low *IFNLR1* expression observed in CD4^+^ T cells could also be modulated with appropriate T cell specific stimulation. In addition, we and others have shown IFN-λs modulate Th2 cytokine production [6, 21, 57], therefore understanding if IFN-λR1 expression changes upon stimulation has direct implications in elucidating mechanistically how Th2 responses are regulated. Only stimulating the TCR with anti-CD3/anti-CD28 antibodies significantly upregulated IFN-λ3 binding to CD4^+^ T cells (Fig 6A). R848 has limited activation potential with human T cells in the absence of other signals [58], therefore R848 activating monocytes and B cells during the 3 day culture did not indirectly upregulate IFN-λ3 binding to CD4^+^ T cells. We further investigated changes in IFN-λR expression at the transcript level in sorted CD4^+^ T cells. We found total *IFNLR1*, but not *IL10RB*, mRNA was significantly increased by TCR stimulation (Fig 6B). Interestingly, only the signaling capable *mIFNLR1*, but not *sIFNLR1*, was upregulated by TCR stimulation (Fig 6C). The *mIFNLR1/sIFNLR1* ratio for CD4^+^ T cells had increased from an average of 2.5 at day 0 to 8.0 at day 3, bringing it closer to ratios we previously observed in epithelial cells. This is the first report of specific regulation of *mIFNLR1* expression by TCR stimulation. We next examined whether TCR upregulation of *IFNLR1* enhanced IFN-λ3 responsiveness. Stimulation of PBMC cultures with anti-CD3/anti-CD28 for 3 days followed by IFN-λ3 addition to sorted CD4^+^ T cells led to enhanced ISG induction for 2 of 3 ISGs tested (Fig 6D). Altogether, our data demonstrate TCR stimulation significantly increases mIFN-λR1 expression on CD4^+^ T cells leading to greater ISG induction in response to IFN-λ3.

### IFN-λ3 treatment inhibits HIV-1 infection of purified CD4^+^ T cells

**Fig 7:**
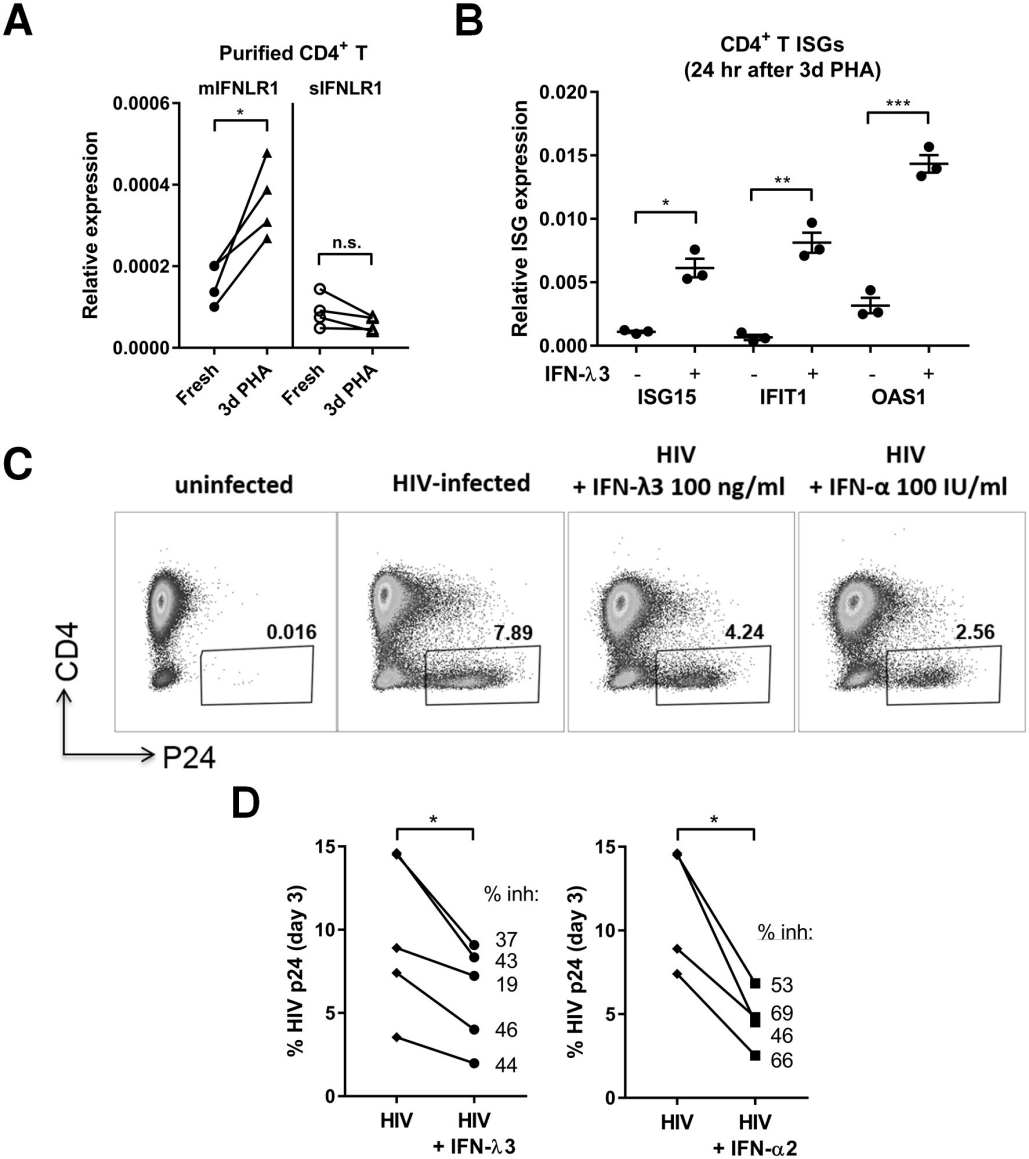
IFN-λ3 inhibits HIV-1 infection of purified CD4^+^ T cells. A) Relative expression of full length, membrane (*mIFNLR1)* and small, soluble (*sIFNLR1*) variant expression in purified CD4^+^ T cells on day 0 or day 3 after PHA stimulation (normalized to *B2M* reference gene). B) ISG (*ISG15*, *IFIT1*, *OAS1*) expression in purified CD4^+^ T cells cultured with or without IFN-λ3 (100 ng/ml) for 24 hrs after 3 days of PHA stimulation. Results are shown as means +/- SD. C-D) Quantification of HIV-1 infection via p24 intracellular flow cytometry. C) Representative p24 staining from 1 healthy individual for all treatments tested. D) % HIV-1 p24 staining from 4–5 different individuals for HIV-1 infection alone compared to cells pre-treated with IFN-λ3 (100 ng/ml) or IFN-α2 (100 IU/ml). Symbols represent means from duplicate wells per individual with each symbol representing a different healthy blood donor. *, P<0.05, **, P<0.01, ***, P<0.001, paired t-tests.

To examine the consequence of increased IFN-λR1 expression on CD4^+^ T cells, we determined if IFN-λ3 could directly inhibit a viral infection of CD4^+^ T cells. We used HIV-1 infection of purified CD4^+^ T cell cultures to monitor whether pre-incubation of IFN-λ3 before HIV-1 inoculation leads to decreased viral infection in the absence of any other immune cells. Optimal HIV-1 infection requires T cell activation [59, 60], therefore we purified total CD4^+^ T cells and activated them with PHA for 3 days. PHA activation upregulated *mIFNLR1* mRNA 2.4 fold on average, but did not upregulate *sIFNLR1* expression (Fig 7A). As expected, subsequent IFN-λ3 treatment of PHA activated CD4^+^ T cells stimulated significant ISG expression compared to no cytokine treated controls (Fig 7B). Next, we tested whether IFN-λ3 induced an antiviral state in PHA-stimulated CD4^+^ T cells. We added IFN-λ3, or pegylated IFN-α2 as our positive control, for 24 hours prior to HIV-1 infection. CD4^+^ T cells treated with either IFN-λ3 or IFN-α2 had significantly decreased HIV-1 p24 positive cells, indicating IFN-λR or IFN-αR signaling inhibited HIV-1 infection (Fig 7C and 7D). These data indicate that type III IFNs can directly impact antiviral responses of peripheral blood CD4^+^ T cells to inhibit HIV-1 infection.

## Discussion

The lack of a sensitive IFN-λR1 antibody has limited our understanding of IFN-λR1 biology in the context of human immune cells. Here, we have clearly identified the main cell types that bind and respond to IFN-λ3 in human peripheral blood, and demonstrate that a unique soluble IFN-λR1 variant present in higher primates inhibits IFN-λ3-mediated ISG induction by binding the cell surface and sequestering IFN-λ3 away from a functional membrane spanning IFN-λR heterodimer complex. Activation of immune cells promotes specific *mIFNLR1* expression and the direct interaction of IFN-λ3 with CD4^+^ T cells induces an antiviral state to decrease HIV-1 infection. Thus, unlike in mice, type III IFNs have the potential to directly impact resting and activated human adaptive immune cells.

Quantification of cell surface IFN-λ3 binding to peripheral blood immune cells correlated with *IFNLR1* transcript expression in most cases. Human B and T cells can interact directly with IFN-λ3, and IFN-λ3 binding levels correlated with the magnitude of ISG induction in stimulation assays. Consistent with previous literature, human monocytes and NK cells did not respond directly to type III IFNs [35, 39, 61]. However, there were exceptions where IFN-λ3 binding did not match *IFNLR1* transcript expression and/or ISG induction. We noted that liver and lung epithelial cells expressed similar or lower total *IFNLR1* mRNA than B cells, but bound ~5-fold more IFN-λ3 and upregulated ISGs up to 50-fold greater than B cells. Upon further analysis of the variants of *IFNLR1*, we found every immune cell isolated expressed greater *sIFNLR1* transcripts relative to *mIFNLR1*, compared to epithelial cells. sIFN-λR1 bound well to every cell type tested, and surprisingly promoted increased binding of IFN-λ3 to the cell surface. Significant inhibition of ISG induction in the presence of sIFN-λR1 likely occurs because the IFN-λ3-sIFN-λR1 complex could not initiate signaling due to the lack of cytoplasmic tail in sIFN-λR1. The lower baseline expression of *sIFNLR1* in epithelial cells, may also be the reason that sIFN-λR1 bound 100% of epithelial cells at a 10–100 fold lower dose than immune cells. Our data add to the understanding of IFN-λ-mediated pathogen control mechanisms by the expression of sIFN-λR1 only in upper primates, and mIFN-λR1 upregulation upon activation of B cells, CD4^+^ T cells, and neutrophils. The presence of the human sIFN-λR1 variant was detected at the time of receptor discovery in 2003 [3, 4], and a previous report indicated pre-incubation of sIFN-λR1 with IFN-λ1 decreased stimulation of a hepatocyte cell line [35], however no mechanisms that changed the relative expression of the 2 main splice forms of IFN-λR1 have been investigated. Soluble cytokine receptors can have either inhibitory or enhancing properties. For example, IL-22BP, also an IL-10 cytokine family member, acts as a decoy blocking IL-22 binding at mucosal surfaces where IL-22R is expressed [62, 63]. In contrast, sIFN-αR2a binding to IFN-α enhances IFN-α responses *in vivo* [64]. It is unclear why primitive primates and lower mammals do not have evidence of sIFN-λR1 expression, while both mice and humans express IL-22BP and sIFN-αR2a. Compared to type I IFNs, type III IFNs induce a slower, lower magnitude response [23, 24] with less inflammatory effects in human clinical trials or in mouse lung infections [25, 65–67]. The expression of sIFN-λR1 may have evolved as a mechanism to contribute to immune cells remaining in an “off” condition until they receive optimal activation signals to allow for increased type III IFN responses, unlike all immune cells responding to type I IFNs at baseline. Since our work identified stimuli that regulate mIFN-λR1, but not sIFN-λR1 expression, determining what stimuli modulate sIFN-λR1 expression will be of future interest.

We observed IFN-λ3 bound well to monocytes despite very little *mIFNLR1* transcript expression, but ISGs were not induced in our assays. The binding was not an artifact of the 6 His-tag on IFN-λ3, since an unrelated, similarly tagged protein did not bind monocytes. IFN-λ3 is not likely binding IL-10RB alone, or high binding would have also been observed to neutrophils, which also express very high levels of IL-10RB. In addition, the affinity of type III IFNs to IL-10RB alone is very low [68]. Since we showed that recombinant sIFN-λR1 directly binds monocytes, we speculate that IFN-λ3 binds monocytes in our assay at least in part because sIFN-λR1 is already bound to the cell surface. Altogether, our data indicate there are multiple layers of regulation of the responses to IFN-λ3.

While many studies have demonstrated potent antiviral activity of type III IFNs at epithelial barrier sites, much less is known regarding type III IFN antiviral activity in peripheral blood. Previous studies proposed that human and mouse T cells do not directly respond to IFN-λs, and that IFN-λ dependent regulatory effects occur via a specific subset of DCs or neutrophils [20, 25, 35, 44, 69–71]. Here, we show for the first time that human CD8^+^ T cells express significantly greater *IFNLR1* transcripts, and bind IFN-λ3 to a higher degree than CD4^+^ T cells, leading to the upregulation of ISGs in response to IFN-λ3 *in vitro*. Only upon TCR or PHA activation of CD4^+^ T cells, did the level of *IFNLR1* mRNA expression match or surpass that seen in CD8^+^ T cells, in turn leading to greater ISG induction and ultimately inhibition of HIV-1 infection by IFN-λ3 treatment. While two previous studies found IFN-λ1 or IFN-λ2 could inhibit or enhance HIV-1 infection of CD4^+^ T cells [72, 73], our findings support the IFN-λ3-mediated induction of an antiviral program in CD4^+^ T cells, with no indication of an enhancement of infection. Our data demonstrate that CD4^+^ and CD8^+^ T cells can be directly regulated by type III IFNs to combat viral infections. While we focused on IFN-λ3 activity in this study, we predict other IFN-λ family members would be able to stimulate ISGs in the same cell types since they all bind the same receptor, but receptor affinity may affect the magnitude of the response [74].

Our results that human neutrophils bind low levels of IFN-λ3 and express low levels of IFN-λR1 are in contrast to mouse models of influenza virus or *Aspergillus fumigatus* infection and arthritis or colitis where mouse neutrophils directly respond to IFN-λ to modulate responses [19, 20, 25, 46]. Limited data has been published measuring ISG induction by IFN-λs in human neutrophils. Human neutrophils responded ~20-fold less than mouse neutrophils to IFN-λ2 [20], and IFIT1 was not induced in human neutrophils after IFN-λ1 treatment [75]. IFN-λ3 may induce greater ISG levels in neutrophils *in vivo* because neutrophils survive longer than the 5 hour time point used in the current study. Additionally, whether human neutrophils are also regulated by the unique STAT-1 independent, non-translational signaling pathway seen after Ifn-λ2 treatment of mouse neutrophils is unclear [20]. Our quantification showing that human neutrophils express low levels of *IFNLR1* transcripts directly *ex vivo* are in agreement with a published human microarray and single cell RNA sequencing results [54, 55]. Subsequent stimulation of neutrophils with TLR agonists and bacteria induced *IFNLR1* mRNA expression, which complements data showing that *Aspergillus fumigatus* addition to neutrophils upregulated IFN-λR1 expression [46]. Therefore, human neutrophils may optimally respond to IFN-λs *in vivo* through upregulation of IFN-λR1 during inflammatory conditions. Future work directly measuring IFN-λR1 protein on the cell surface after stimulations will only be possible when a specific, sensitive antibody becomes available.

There is a growing list of differences between the mouse and human type III IFN system. Mice only have functional *Ifnl2* and *Ifnl3* genes, and here we show the sIFN-λR1 splice variant is not present in mice and other non-primates. Although initial studies found human B cells do not respond to IFN-λs [35, 39], we and another group have now clearly demonstrated human B cells directly respond to IFN-λ3 (current study) and IFN-λ1 [45]. Our data showing IFN-λ3 binds to human pDCs matches previous studies showing human pDCs respond to type III IFNs [41, 43, 44], but studies have found contradicting results for whether type III IFNs stimulate ISGs in mouse pDCs [7, 20]. We showed that activation through TCR, BCR, or TLRs upregulated *IFNLR1* mRNA expression and IFN-λ3 binding to multiple human immune cell types, but a recent study found TLR3 stimulation or addition of Sendai virus did not upregulate *IFNLR1* transcript expression in a hepatocyte cell line [26]. Therefore, *IFNLR1* expression may be differentially regulated in epithelial cells compared to immune cells. Interestingly, we found IFN-α2 decreased IFN-λ3 binding to B cells, whereas a previous study demonstrated IFN-α treatment upregulates *IFNLR1* mRNA expression in hepatocytes [9]. IFN-γ treatment alone was also unable to upregulate IFN-λR1 expression in B cells or T cells. Our findings demonstrate IFN-λR1 expression is uniquely regulated in immune cells compared to epithelial cells, and future work should confirm if IFN-λR1-dependent regulatory mechanisms discovered in mouse models are reproducible in human studies.

In summary, our study has provided clear evidence of which human immune cells express functional IFN-λR with major differences in IFN-λR1 expression between mice and humans, especially within the adaptive immune system. The remarkable absence of IFN-λR1 expression in most mouse immune cells begs the question for how phenotypes and immunoregulatory mechanisms could differ during a viral infection or autoimmune mouse model if IFN-λR1 cell expression mimicked that seen in humans. Going forward, studying the regulation of human IFN-λR1 expression in epithelial cells and immune cells will provide critical information to guide mechanistic studies related to type III IFN regulation of immune responses, with the goal of developing therapies to fight viruses, dampen chronic inflammation at mucosal sites, and treat cancer with less side effects compared to type I IFNs.

## Materials and methods

### Ethics statement

This study was approved by the University of Alberta Health Research Ethics Board (Pro00046564). All blood donors gave written informed consent in accordance with the Declaration of Helsinki. Normal human lungs that were not used for transplantation were obtained via a tissue retrieval service (International Institute for the Advancement of Medicine, Edison, NJ). The identity of donors was not provided, although basic demographic data was included. Ethical approval to obtain normal human bronchial epithelial cells (NHBE) was obtained from the Conjoint Health Research Ethics Board of the University of Calgary and from the Internal Ethics Board of the International Institute for the Advancement of Medicine.

### Cell lines and primary cell isolation

Huh7.5 cells (from Dr. Charles Rice, The Rockefeller University) and Huh7 IFNLR1 knockout cells [49] (from Dr. Ram Savan, University of Washington), were cultured in DMEM containing: 10% fetal bovine serum (FBS), 100 U/ml penicillin, 100 μg/ml streptomycin, 1X MEM non-essential amino acids and 4 mM L-glutamine (media from GE Healthcare, supplements from Thermofisher). BEAS-2B and DG75 cells were obtained from ATCC and cultured using the BEGM bronchial epithelial growth medium Bulletkit (Lonza) according to the manufacturer’s instructions, except hydrocortisone was not added to medium during any cytokine stimulations. PBMCs were isolated from healthy human individuals by Ficoll-Paque PLUS (GE Healthcare) gradient centrifugation. All PBMC experiments used freshly isolated cells. Immune cells were sorted using a BD FACSAria III cell sorter within a biosafety cabinet with dead cells excluded using LIVE/DEAD Near IR (Thermofisher) after gating out doublets. Cells were sorted as follows: B cells: CD20^+^CD3^-^CD56^-^CD14^-^, CD4^+^ T cells: CD3^+^CD4^+^CD8^-^, CD8^+^ T cells: CD3^+^CD8^+^CD4^-^, Monocytes: CD14^+^CD3^-^CD20^-^, NK cells: CD56^+^CD3^-^. Purities were routinely ≥ 97–99% and pDCs were not detectable by flow cytometry (CD123^+^HLA-DR^+^). Representative purity results are shown in S5 Fig. In some experiments, B cells were isolated with a negative B cell isolation kit (StemCell Technologies) or CD14^+^ monocytes were isolated by CD14 positive selection (Miltenyi Biotec), but results were comparable whether cells were isolated via FACS or magnetic selection. PBMCs or isolated immune cells were cultured in RPMI 1640 containing: 10% FBS, 100 U/ml penicillin, 100 μg/ml streptomycin, 10 mM HEPES and 2 mM glutamax (media from GE Healthcare, supplements from Thermofisher). Primary human hepatocytes were purchased from BioreclamationIVT. Normal, nontransplanted human lungs were obtained via a tissue retrieval service (International Institute for the Advancement of Medicine, Edison, NJ). Bronchial epithelial cells were isolated as previously described [76], and cultured in BEGM with supplements (Lonza) as previously described [77]. In brief, cells were plated in 6 or 12 well plates and utilized at ~70% confluence (typically after 10–11 days with media change every 2 days) for binding or stimulation assays. Neutrophils were isolated from healthy donor whole blood with Polymorphprep (Axis Shield) according to the manufacturer’s protocol to achieve highly pure, unprimed neutrophils suitable for gene expression studies [78]. Purities were ≥ 95% (S2 Fig) and pDCs were not detectable by flow cytometry. IFN-λ3 binding to neutrophils was also quantified after RBC magnetic depletion (StemCell Technologies). All cells were maintained in an incubator with a humidified atmosphere with 5% CO_2_ at 37°C.

### Flow cytometry

The IFN-λ3 binding assay was performed as published [48]. In brief, cells were cultured with or without His-tagged IFN-λ3 (R&D Systems) diluted in PBS containing 1% BSA on ice for 60 min at indicated doses. In some experiments, recombinant sIFN-λR1 or IL-10RB protein (R&D Systems) was added simultaneously with IFN-λ3 while FcγRs were blocked with 150 μg/ml human IgG (Jackson Immunoresearch) or human Fc Block (BD Biosciences). Cells were then washed (PBS + 1% BSA + 0.05% sodium azide) and stained with anti-His PE (Miltenyi Biotec) in combination with multiple surface marker antibodies on ice for 40 min in the dark. Anti-His PE secondary antibody was added alone in a parallel sample to obtain background fluorescence that was subtracted from each sample (‘background subtracted’), and background was always less than 1%. Cells were washed again and re-suspended in 2% paraformaldehyde (Electron Microscopy Sciences) at room temperature for 15 min in the dark. Paraformaldehyde was washed away prior to analysis. sIFN-λR1-Fc or IL-10RB-Fc (R&D Systems) binding to the cell surface was similarly quantified. sIFN-λR1 or IL-10RB was added to cells for 45 min on ice and after washing, anti-Fc PE (Biolegend) was added to detect receptor bound to the cell surface. anti-Fc PE antibody was always added alone in a parallel sample so that any background would be subtracted. The following antibodies were used to identify our subpopulations: CD3 (clone UCHT1 Biolegend), CD4 (clone SK3 BD Bioscience, RPA-T4 eBioscience), CD8 (clone SK1 Biolegend), CD14 (clone HCD14 Biolgend, 61D3 eBioscience), CD56 (clone HCD56 Biolegend), CD20 (clone 2H7 Biolegend), CD66b (clone G10F5 Biolegend), HLA-DR (clone L243 eBioscience), CD123 (clone 6G6 eBioscience), CD11c (clone Bu15 Biolegend), CD16 (clone 3G8 Biolegend), IgD (clone IA6-2 Biolegend), CD27 (clone O323 Biolegend). For each antibody panel, a dump channel, where antibodies to multiple immune cells not of interest for that assay, was used to exclude contaminating cell types. Examples of our gating strategy are shown in S1 and S2 Figs. For stimulation experiments, LIVE/DEAD Near IR (Thermofisher) was utilized within the dump gate to determine live cells for IFN-λ3 binding quantification. NHBE and hepatocytes were incubated with IFN-λ3 in the same binding assay without any surface staining antibodies since they were pure populations. Samples were analyzed using a BD LSR Fortessa X-20 or LSR Fortessa-SORP Flow Cytometer (5 laser: 375 nm, 405 nm, 488 nm, 561 nm, and 633 nm) and FlowJo software (BD Biosciences) was used for data analysis and graph generation. In certain experiments, sIFN-λR1 binding was also visualized via imaging cytometry using the Amnis ImageStream^®^X mark II Flow Cytometer (Millipore-Sigma) and Inspire^®^ (Amnis) software. 6,000–12,000 total events were collected for each experiment. Samples were imaged at 60x magnification and IDEAS^®^ (Amnis) software was used for single cell analysis.

### Real-time reverse transcription PCR (RT-qPCR)

PBMCs or sorted immune cell subsets (2 x 10^6^ cells/ml) or NHBE (6 well plate, 70% confluence) were incubated at 37°C with or without IFN-λ3 (100 ng/ml) or other stimuli for the time points indicated. Stimuli tested in this study include: IFN-λ3 (R&D Systems), IFN-α2b (INTRON^®^ A, Merck), recombinant IFN-λR1 and IL-10RB (R&D Systems), R848 (Invivogen), anti-CD3 (clone HIT3a) and anti-CD28 (clone CD28.2) were from Biolegend. Anti-IgM, IgG, IgA (Jackson Immunoresearch), anti-CD40 (R&D Systems), IFN-γ (Peprotech). Heat killed *Listeria monocytogenes* 10403S supernatant was prepared as described [79]. Cells were then washed with PBS and the pellet was resuspended in TRIzol (Thermofisher). For quantifying *IFNLR1* and *IL10RB* transcripts directly *ex vivo*, sorted cells were resuspended in TRIzol without incubation. All samples were stored at -80°C. Total RNA was extracted with Direct-zol mini or micro RNA isolation kits (Zymo Research) in accordance with manufacturer’s guidelines, with on column DNase I digestion. Reverse transcription was performed with a Superscript VILO IV mastermix (Thermofisher). RT-qPCR was performed with a Bio-Rad CFX 96 using Thermofisher POWER SYBR mastermix (Thermofisher) according to the manufacturer’s protocol: 95°C for 10 min, and then repeating 40 times, 95°C for 15 sec and 60°C for 60 sec, followed by a melting curve analysis. SYBR green primer sequences are listed in S4 Table, with the exception of the *B2M* QuantiTect Primers (Qiagen). For Taqman analysis of *IFNLR1* (Hs00417120_m1), *IL10RB* (Hs00175123_m1), *HPRT1* (Hs02800695_m1), *RPL13A* (Hs04194366_g1) and *B2M* (Hs99999907_m1) (Thermofisher), Taqman Fast Advanced mastermix (Thermofisher) was used with the following cycling parameters: 50°C for 2 min, 95°C for 2 min, and then repeating 40 times, 95°C for 1 sec and 60°C for 20 sec. No template controls were run for every set of primers on each plate. Samples were normalized to the geometric mean of two reference genes, *HPRT1* and *RPL13A*, as described [80], unless indicated when *B2M* was the reference gene that was stably expressed between samples. Fold changes in mRNA expression were calculated using the ΔΔCt method with comparisons of stimulated to unstimulated cells. Relative expression values were calculated by 2^-(ΔCT)^ after normalization to reference genes indicated.

### CD4^+^ T cell HIV-1 infection

CD4^+^ T cells were isolated using a CD4^+^ T cell enrichment kit (StemCell Technologies) according to manufacturer’s instructions. The enriched CD4^+^ T cells (2×10^6^ cells/ml) were maintained in RPMI 1640 media supplemented with 10% FBS, 100 U/ml penicillin, 100 μg/ml streptomycin, 10 μg/ml phytohemagglutinin-M (PHA-M; Sigma), and 50 U/ml recombinant IL-2 at 37°C and 5% CO_2_ incubator for 3 days. Excess PHA was removed by washing with fresh media and cell density was adjusted to 7×10^5^ cells/ml in 48 well plates. Activated T cells were cultured with media alone, IFN-α2 (100 IU/ml) or IFN-λ3 (100 ng/ml) for 24 hours before CXCR4-tropic HIV-1 LAI virus (AIDS Research and Reagent Program, NIH) infection via magnetofection (OZ Biosciences), as previously described [81]. Non-bound, excess HIV-1 virus was washed away after 24 hrs of infection and fresh media with or without IFNs were added back for an additional 2 days in culture. Uninfected and infected CD4^+^ T cells with or without IFN-λ3 treatment were stained intracellularly with HIV-1 KC57-p24 core (Beckman Coulter) antibody on day 3 post HIV-1 infection.

### Statistics and data analysis

Graphs were formulated and data were analyzed in Graphpad Prism 7 or FlowJo software (BD Biosciences). A P-value less than 0.05 was considered significant. The number of healthy donors or replicates and statistical tests are specified in each figure legend.

## Supporting information

S1 Fig Flow cytometry gating strategy. A) Gating total cells and removing any doublets. B-H) Outline of gating strategy for B cells (B), monocytes (C), natural killer (NK) cells (D), plasmacytoid dendritic cells (pDCs) (E), CD4^+^ T cells (F), myeloid DCs (mDCs) (G) and CD8^+^ T cells (H). Dump refers to multiple antibodies labeled with same fluorophore added to exclude other subsets (eg. Dump gate for B cells: antibodies to CD3, CD14, CD56 and CD16).
(TIF)

S2 Fig Flow cytometry gating strategy for freshly isolated neutrophils. Neutrophils purified with Polymorphprep gradient centrifugation were identified as CD66b^+^ CD16^+^ after gating by size and gating out T cells (CD3), B cells (CD20) and monocytes (CD14). Purities were routinely >95–99%.
(TIF)

S3 Fig Epithelial cells bind greater levels of IFN-λ3 than immune cells, results are reproducible over time, and IFN-λ3 binding requires *IFNLR1* expression. A-B) IFN-λ3 binding was quantified via flow cytometry as described in the Materials and methods. A) Fold increase in median PE binding after adding 1 or 5 μg/ml IFN-λ3 to epithelial cells (NHBE or hepatocytes (hep)) or total human PBMCs with gating on B cells, monocytes (mono), pDCs or mDCs. Graph shows mean +/- SD for 3 (hep), 5 (NHBE), 8–14 (1 μg/ml immune cell) or 21–22 (5 μg/ml immune cell) different donors. B) The % IFN-λ3+ cells quantified for monocytes (mono) or B cells from our binding assay repeated on the same healthy individual at least 6 months apart. C) Binding percentages to CD3^+^ T cells as detected by flow cytometry for IFN-λ3 or a control protein that was similarly his-tagged (OBCAM) where means +/- SD are shown. Each symbol represents a different individual. D) IFN-λ3 binding to Huh7 *IFNLR1* knockout cells compared to adding the secondary antibody alone. Data are representative of 2 independent experiments.
(TIF)

S4 Fig IFN-λ3 binding levels significantly correlate between specific immune cell subsets. Pearson correlation coefficients (r) calculated when comparing IFN-λ3 percent binding to immune cell subsets where each symbol is a different healthy individual.
(TIF)

S5 Fig Purity of cells after sorting. Representative flow cytometry plots of cells acquired after sorting checking the purity of the populations we used for RT-qPCR.
(TIF)

S6 Fig Baseline ISG expression and IFN-α2 mediated ISG induction in purified primary human cells. A) Baseline (untreated) expression levels of *ISG15*, *IFIT1* and *OAS1* in isolated cell types. B) RT-qPCR quantification of *ISG15*, *IFIT1*, and *IFI44* induced after addition of positive control IFN-α2 (1000 IU/ml (neutrophil), 100 IU/ml (monocyte, B cell, CD4^+^ or CD8^+^ T cells)) to purified cells. Neutrophils were treated for 5 hrs, all other cell types were treated for 24 hrs. Graphs show relative expression (A) or fold induction relative to unstimulated negative control (B) after normalization to the geomean of *HPRT1* and *RPL13A* reference genes. Bars represent mean + SEM from 4–6 (B, T cell), 3–4 (monocyte), 4–6 (neutrophil) or 5 normal human bronchial epithelial cell (NHBE) different donors. *, P<0.05, **, P<0.01, ***, P<0.001, ****, P<0.0001, one-way ANOVA, Tukey’s multiple comparisons test where significant comparisons to monocytes (mono, m) and neutrophils (neut, n) are shown (A). All other comparisons were not significant.
(TIF)

S7 Fig Soluble IFN-λR1 directly binds to Huh7.5 cells and enhances IFN-λ3 binding. A) Quantification of recombinant sIFN-λR1 (0.01, 0.1 μg/ml) binding to Huh7.5 cells with or without IFN-λ3 (100 ng/ml). B) IFN-λ3 binding to Huh7.5 cells where IFN-λ3 (0.1 μg/ml) was added with or without sIFN-λR1 (0.1, 1 μg/ml) or IL-10RB (1 μg/ml). C) IFN-λ3 (0.25 μg/ml) binding to Huh7.5 cells when added alone or with sIFN-λR1 (0.5 μg/ml) added either simultaneously or sIFN-λR1 was added first for 45 min on ice before cells were washed twice and then IFN-λ3 added. A-C) Histograms are representative of 2–3 independent experiments. 2^nd^ antibody (Ab) alone is negative control to show background fluorescence: A) anti-Fc PE alone, B-C) anti-his PE alone.
(TIF)

S1 Table Statistical analyses comparing IFN-λ3 binding between immune cell subsets and NHBE. 5 μg/ml IFN-λ3 binding results were compared in 3–22 different individuals. One-way ANOVA with Tukey’s multiple comparisons. n.s. = not significant, *, P<0.05, **, P<0.01, ***, P<0.001, ****, P<0.0001. Data relates to Fig 1D.
(DOCX)

S2 Table Correlation coefficients of percent IFN-λ3 binding between immune cell subsets. Pearson correlation coefficients (P value result in brackets, n.s. = not significant, *, P<0.05, ***, P<0.001) calculated from 5 μg/ml IFN-λ3 binding results from 11–18 different individuals.
(DOCX)

S3 Table Evidence for the presence of a small/soluble variant of *IFNLR1* across multiple species. (DOCX)

S4 Table List of SYBR RT-qPCR primer sequences. (DOCX)
