# Supplementary figures and images for: Differential expression of interferon-lambda receptor 1 splice variants determines the magnitude of the antiviral response induced by interferon-lambda 3 in human immune cells
Source: PLoS Pathog. 2020 Apr 30;16(4):e1008515. doi: 10.1371/journal.ppat.1008515 (PMC7217487; doi:10.1371/journal.ppat.1008515)

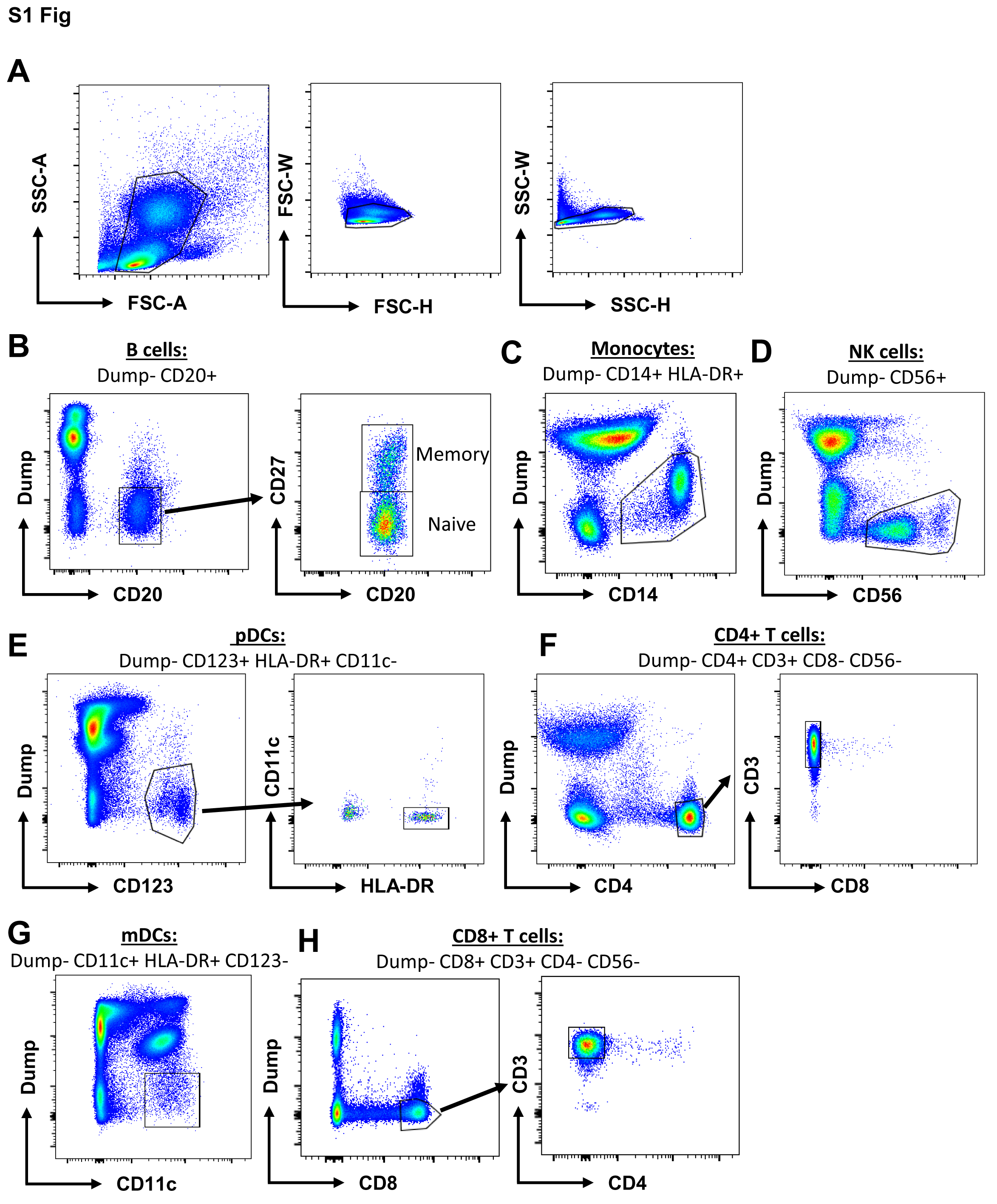

Supplement: S1 Fig — A) Gating total cells and removing any doublets. B-H) Outline of gating strategy for B cells (B), monocytes (C), natural killer (NK) cells (D), plasmacytoid dendritic cells (pDCs) (E), CD4+ T cells (F), myeloid DCs (mDCs) (G) and CD8+ T cells (H). Dump refers to multiple antibodies labeled with same fluorophore added to exclude other subsets (eg. Dump gate for B cells: antibodies to CD3, CD14, CD56 and CD16). (TIF) [file ppat.1008515.s001.tif]

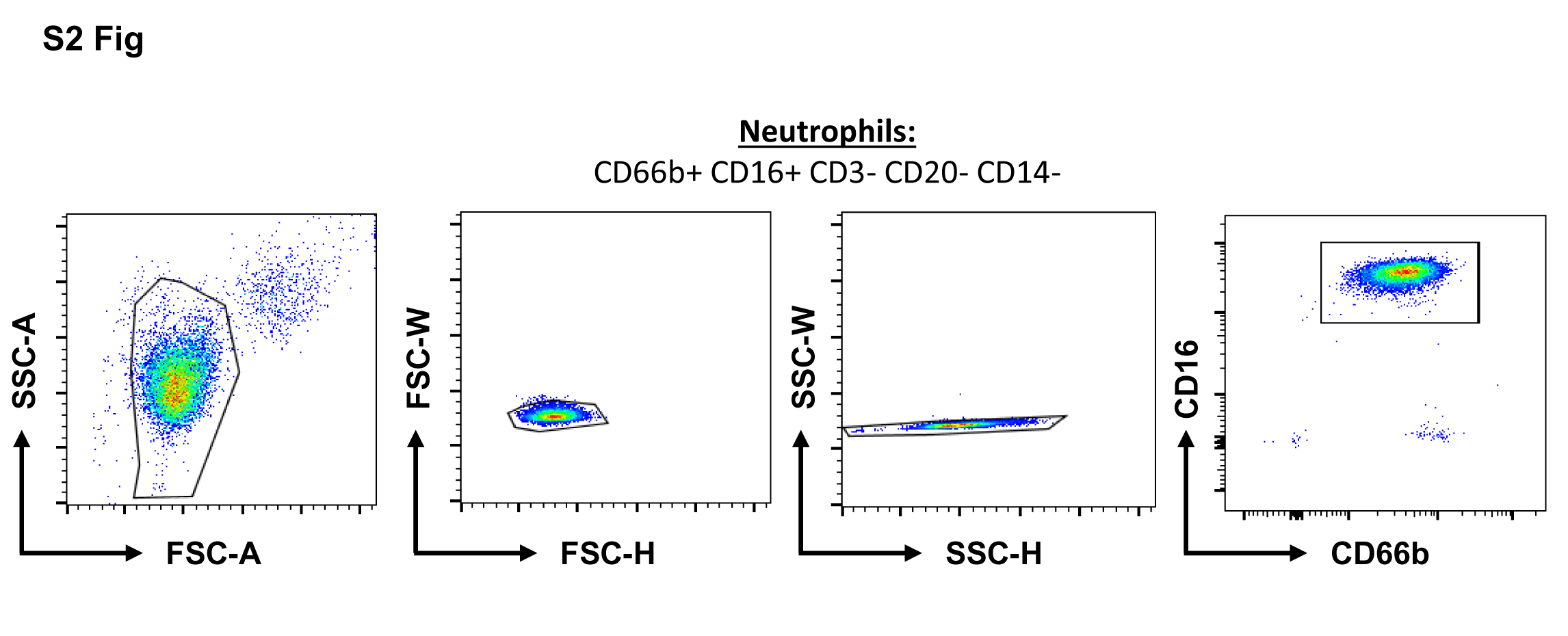

Supplement: S2 Fig — Neutrophils purified with Polymorphprep gradient centrifugation were identified as CD66b+ CD16+ after gating by size and gating out T cells (CD3), B cells (CD20) and monocytes (CD14). Purities were routinely >95–99%. (TIF) [file ppat.1008515.s002.tif]

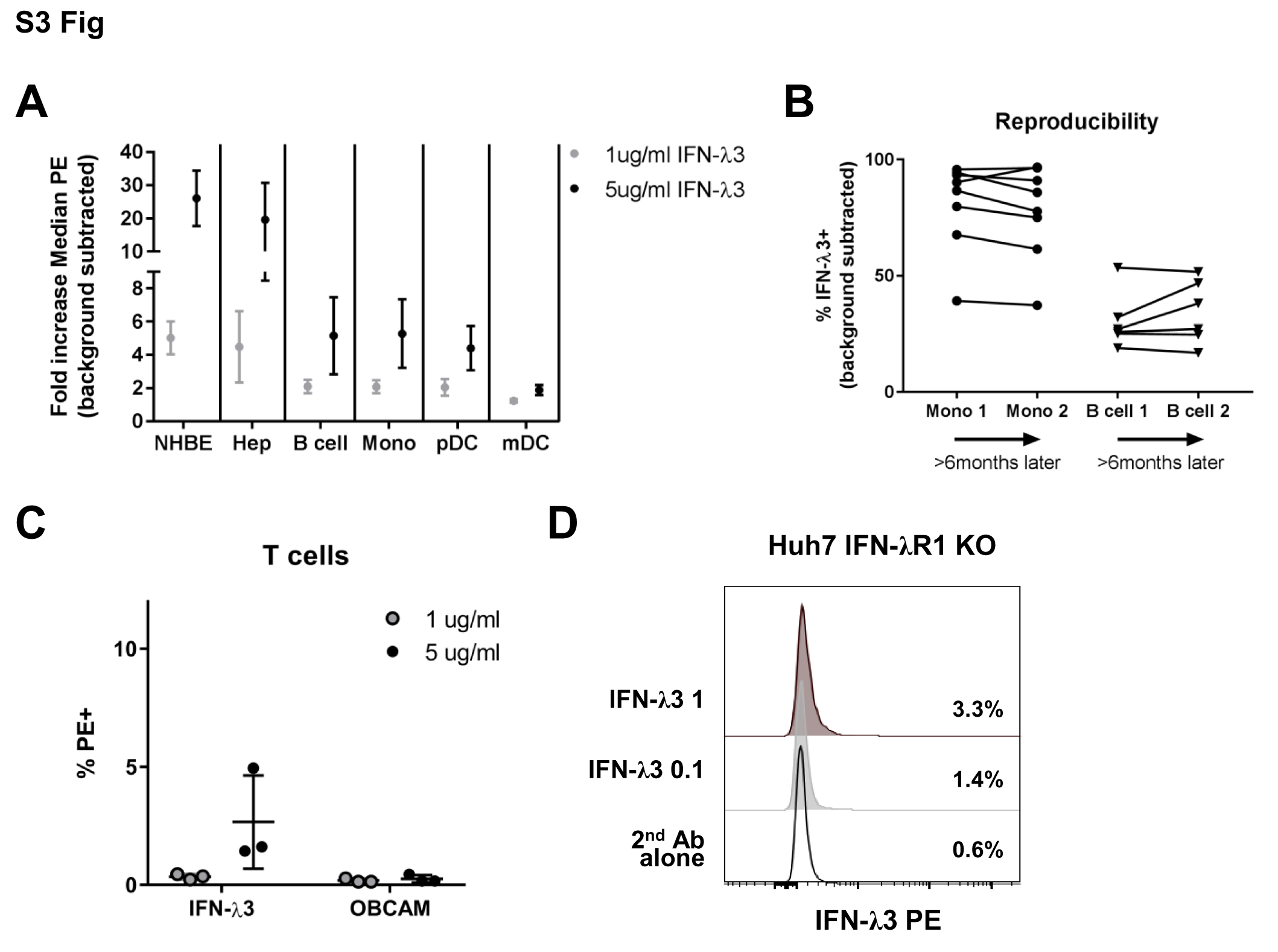

Supplement: S3 Fig — A-B) IFN-λ3 binding was quantified via flow cytometry as described in the Materials and methods. A) Fold increase in median PE binding after adding 1 or 5 μg/ml IFN-λ3 to epithelial cells (NHBE or hepatocytes (hep)) or total human PBMCs with gating on B cells, monocytes (mono), pDCs or mDCs. Graph shows mean +/- SD for 3 (hep), 5 (NHBE), 8–14 (1 μg/ml immune cell) or 21–22 (5 μg/ml immune cell) different donors. B) The % IFN-λ3+ cells quantified for monocytes (mono) or B cells from our binding assay repeated on the same healthy individual at least 6 months apart. C) Binding percentages to CD3+ T cells as detected by flow cytometry for IFN-λ3 or a control protein that was similarly his-tagged (OBCAM) where means +/- SD are shown. Each symbol represents a different individual. D) IFN-λ3 binding to Huh7 IFNLR1 knockout cells compared to adding the secondary antibody alone. Data are representative of 2 independent experiments. (TIF) [file ppat.1008515.s003.tif]

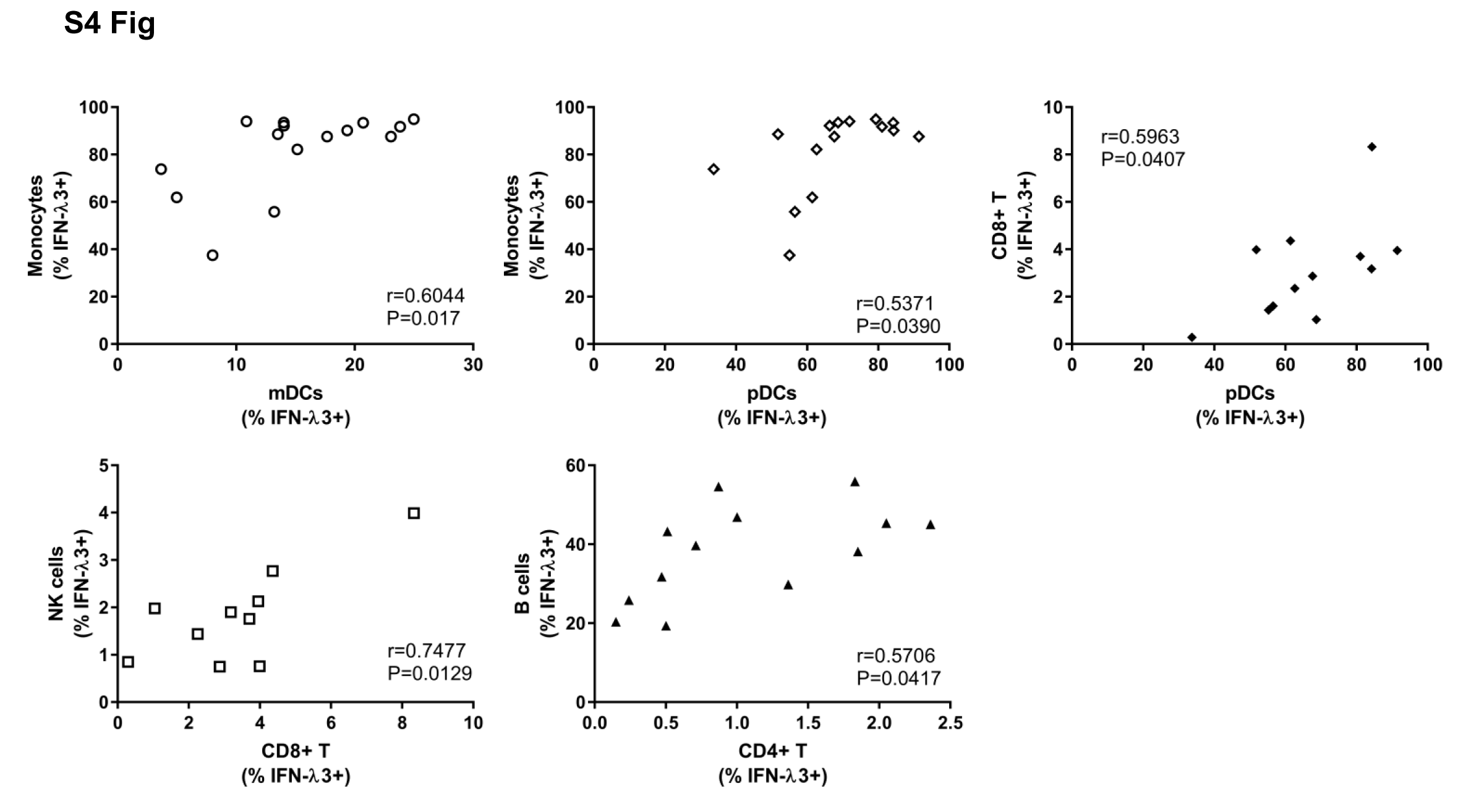

Supplement: S4 Fig — Pearson correlation coefficients (r) calculated when comparing IFN-λ3 percent binding to immune cell subsets where each symbol is a different healthy individual. (TIF) [file ppat.1008515.s004.tif]

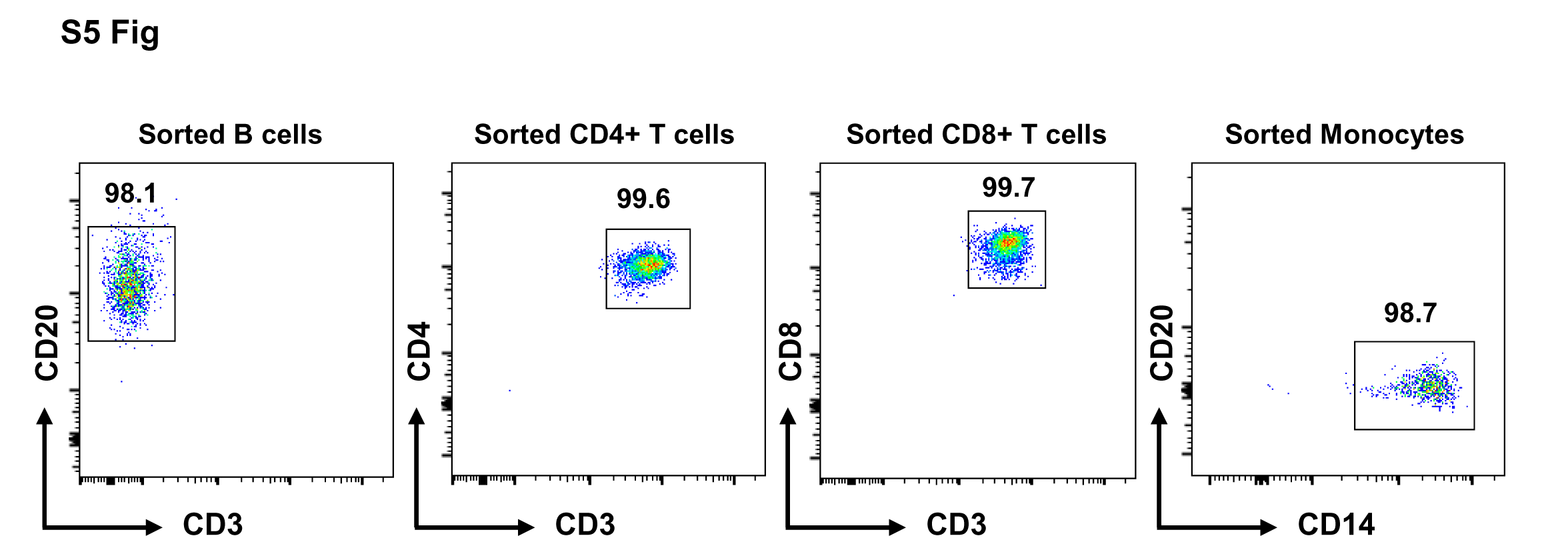

Supplement: S5 Fig — Representative flow cytometry plots of cells acquired after sorting checking the purity of the populations we used for RT-qPCR. (TIF) [file ppat.1008515.s005.tif]

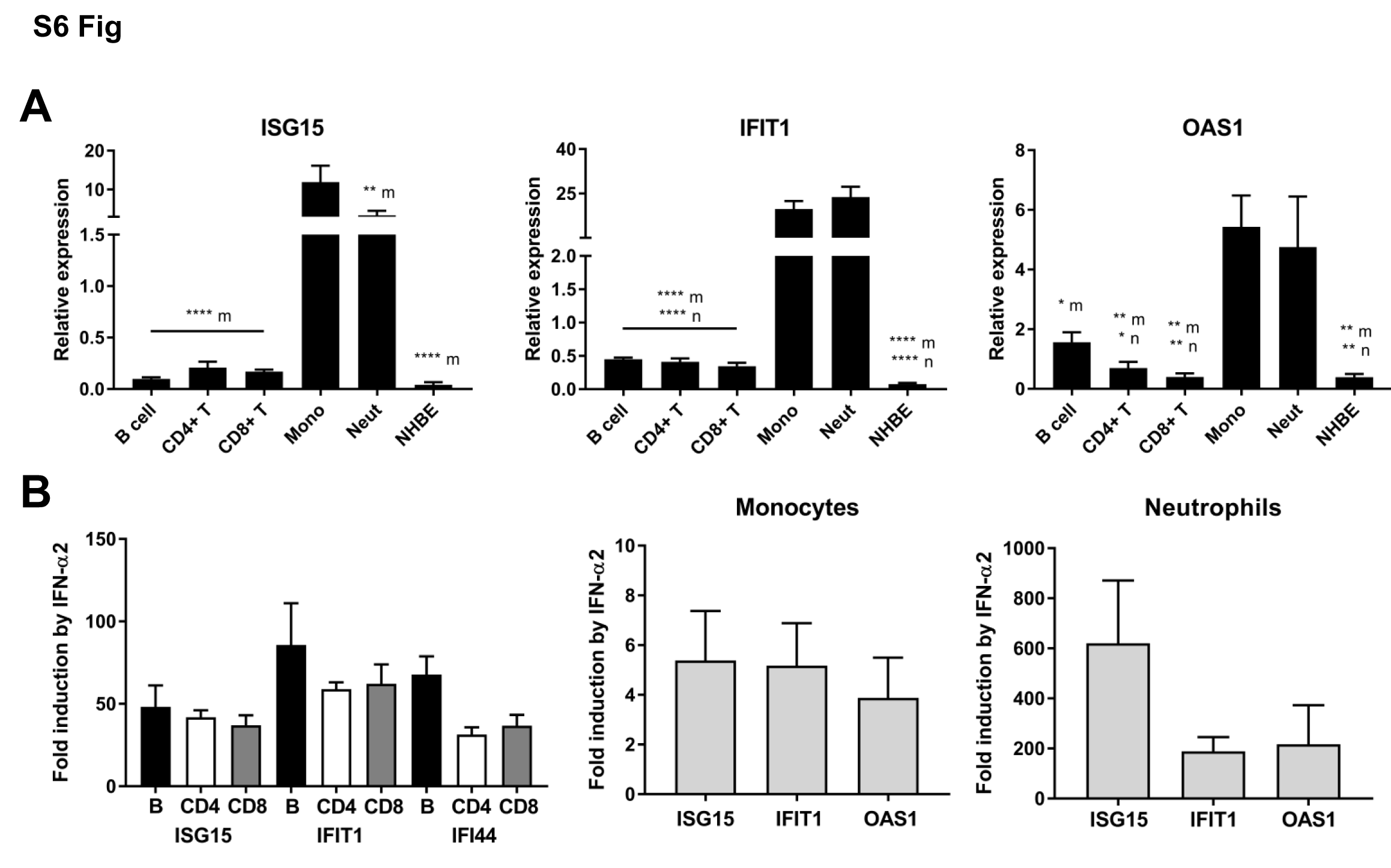

Supplement: S6 Fig — A) Baseline (untreated) expression levels of ISG15, IFIT1 and OAS1 in isolated cell types. B) RT-qPCR quantification of ISG15, IFIT1, and IFI44 induced after addition of positive control IFN-α2 (1000 IU/ml (neutrophil), 100 IU/ml (monocyte, B cell, CD4+ or CD8+ T cells)) to purified cells. Neutrophils were treated for 5 hrs, all other cell types were treated for 24 hrs. Graphs show relative expression (A) or fold induction relative to unstimulated negative control (B) after normalization to the geomean of HPRT1 and RPL13A reference genes. Bars represent mean + SEM from 4–6 (B, T cell), 3–4 (monocyte), 4–6 (neutrophil) or 5 normal human bronchial epithelial cell (NHBE) different donors. *, P<0.05, **, P<0.01, ***, P<0.001, ****, P<0.0001, one-way ANOVA, Tukey’s multiple comparisons test where significant comparisons to monocytes (mono, m) and neutrophils (neut, n) are shown (A). All other comparisons were not significant. (TIF) [file ppat.1008515.s006.tif]

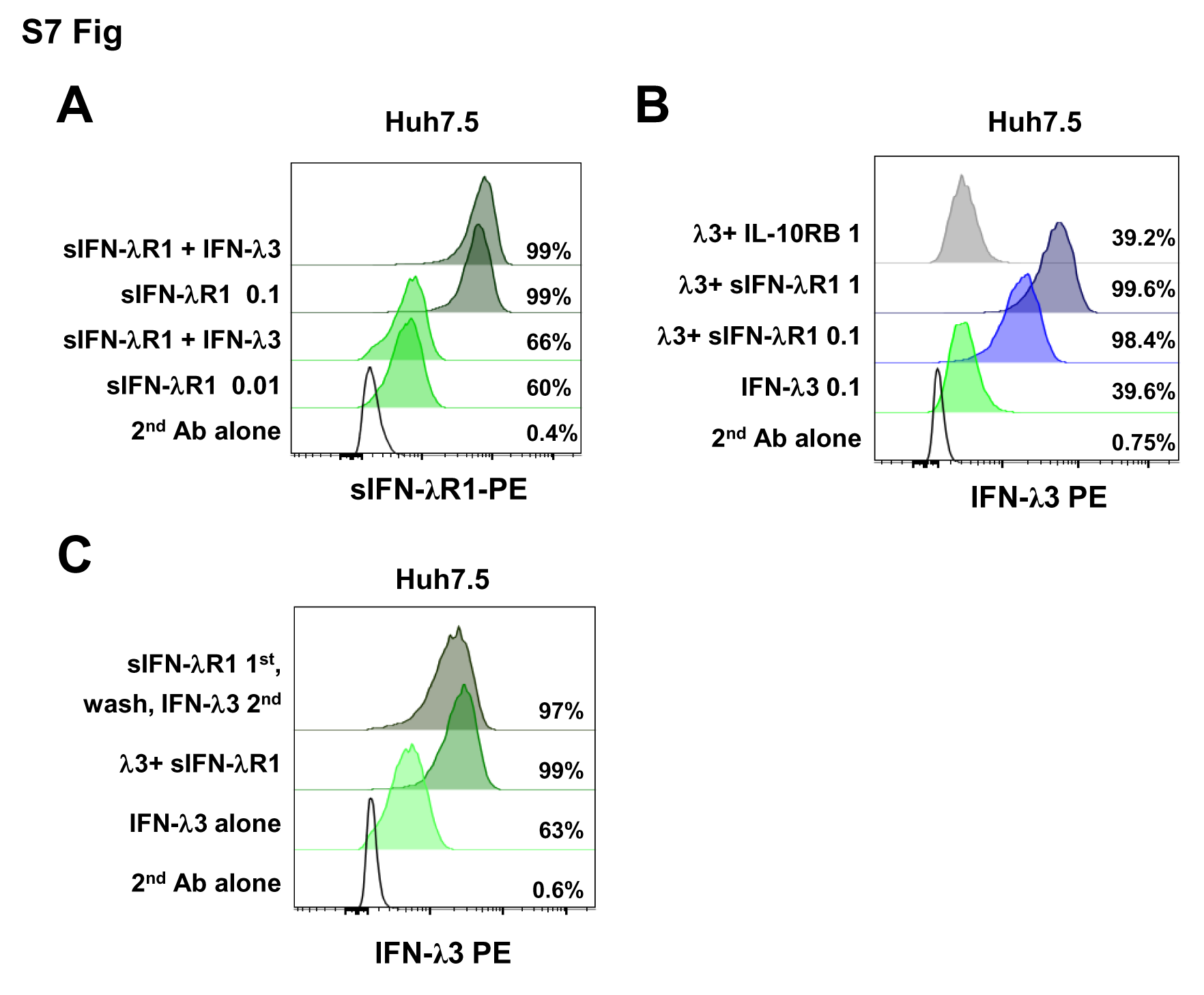

Supplement: S7 Fig — A) Quantification of recombinant sIFN-λR1 (0.01, 0.1 μg/ml) binding to Huh7.5 cells with or without IFN-λ3 (100 ng/ml). B) IFN-λ3 binding to Huh7.5 cells where IFN-λ3 (0.1 μg/ml) was added with or without sIFN-λR1 (0.1, 1 μg/ml) or IL-10RB (1 μg/ml). C) IFN-λ3 (0.25 μg/ml) binding to Huh7.5 cells when added alone or with sIFN-λR1 (0.5 μg/ml) added either simultaneously or sIFN-λR1 was added first for 45 min on ice before cells were washed twice and then IFN-λ3 added. A-C) Histograms are representative of 2–3 independent experiments. 2nd antibody (Ab) alone is negative control to show background fluorescence: A) anti-Fc PE alone, B-C) anti-his PE alone. (TIF) [file ppat.1008515.s007.tif]
