# Supplementary material for: Differential expression of interferon-lambda receptor 1 splice variants determines the magnitude of the antiviral response induced by interferon-lambda 3 in human immune cells
Source: PLoS Pathog. 2020 Apr 30;16(4):e1008515. doi: 10.1371/journal.ppat.1008515 (PMC7217487; doi:10.1371/journal.ppat.1008515)
Supplement: S1 Table — 5 μg/ml IFN-λ3 binding results were compared in 3–22 different individuals. One-way ANOVA with Tukey’s multiple comparisons. n.s. = not significant, *, P<0.05, **, P<0.01, ***, P<0.001, ****, P<0.0001. Data relates to Fig 1D. (DOCX) [file ppat.1008515.s008.docx]

**Table S1: Statistical analyses comparing IFN-λ3 binding between immune cell subsets and NHBE**

|  | **NHBE** | **Hep** | **B cell** | **T cell** | **Mono** | **NK** | **Neut** | **pDC** | **mDC** |
| --- | --- | --- | --- | --- | --- | --- | --- | --- | --- |
| **NHBE** |  |  |  |  |  |  |  |  |  |
| **Hep** | **n.s.** |  |  |  |  |  |  |  |  |
| **B cell** | ******** | ******** |  |  |  |  |  |  |  |
| **T cell** | ******** | ******** | ******** |  |  |  |  |  |  |
| **Mono** | ***** | ***** | ******** | ******** |  |  |  |  |  |
| **NK** | ******** | ******** | ******** | **n.s.** | ******** |  |  |  |  |
| **Neut** | ******** | ******** | ******** | **n.s.** | ******** | **n.s.** |  |  |  |
| **pDC** | ******** | ******** | ******** | ******** | ****** | ******** | ******** |  |  |
| **mDC** | ******** | ******** | ******** | ******* | ******** | ****** | ***** | ******** |  |

5 μg/ml IFN-λ3 binding results were compared in 3-22 different individuals. One-way ANOVA with Tukey’s multiple comparisons. n.s. = not significant, *, P<0.05, **, P<0.01, ***, P<0.001, ****, P<0.0001. Data relates to **Fig 1D**.
