# Supplementary material for: Differential expression of interferon-lambda receptor 1 splice variants determines the magnitude of the antiviral response induced by interferon-lambda 3 in human immune cells
Source: PLoS Pathog. 2020 Apr 30;16(4):e1008515. doi: 10.1371/journal.ppat.1008515 (PMC7217487; doi:10.1371/journal.ppat.1008515)
Supplement: S2 Table — Pearson correlation coefficients (P value result in brackets, n.s. = not significant, *, P<0.05, ***, P<0.001) calculated from 5 μg/ml IFN-λ3 binding results from 11–18 different individuals. (DOCX) [file ppat.1008515.s009.docx]

**Table S2: Correlation coefficients of percent IFN-λ3 binding between immune cell subsets**

|  | **pDC** | **mDC** | **B cell** | **CD4+ T cell** | **CD8+ T cell** | **Monocyte** | **NK cell** | **Neutrophil** |
| --- | --- | --- | --- | --- | --- | --- | --- | --- |
| **pDC** |  |  |  |  |  |  |  |  |
| **mDC** | **0.8364 (***)** |  |  |  |  |  |  |  |
| **B cell** | 0.3123 (n.s.) | 0.1179 (n.s.) |  |  |  |  |  |  |
| **CD4+ T cell** | 0.4249 (n.s.) | 0.2858 (n.s.) | **0.5706 (*)** |  |  |  |  |  |
| **CD8+ T cell** | **0.5963 (*)** | 0.4353 (n.s.) | 0.4233 (n.s.) | **0.8651 (***)** |  |  |  |  |
| **Monocyte** | **0.5371 (*)** | **0.6044 (*)** | -0.0385 (n.s.) | 0.4006 (n.s.) | 0.2344 (n.s.) |  |  |  |
| **NK cell** | 0.4249 (n.s.) | -0.0103 (n.s.) | 0.2102 (n.s.) | 0.6105 (n.s P=0.06) | **0.7477 (*)** | 0.1040 (n.s.) |  |  |
| **Neutrophil** | -0.4976 (n.s.) | -0.4296 (n.s.) | -0.0749 (n.s.) | -0.2349 (n.s.) | -0.3285 (n.s.) | -0.0047 (n.s.) | 0.4388 (n.s.) |  |

Pearson correlation coefficients (P value result in brackets, n.s. = not significant, *, P<0.05, ***, P<0.001) calculated from 5 μg/ml IFN-λ3 binding results from 11-18 different individuals.
