# Supplementary material for: Differential expression of interferon-lambda receptor 1 splice variants determines the magnitude of the antiviral response induced by interferon-lambda 3 in human immune cells
Source: PLoS Pathog. 2020 Apr 30;16(4):e1008515. doi: 10.1371/journal.ppat.1008515 (PMC7217487; doi:10.1371/journal.ppat.1008515)
Supplement: S3 Table — (DOCX) [file ppat.1008515.s010.docx]

**Table S3: Evidence for the presence of a small/soluble variant of *IFNLR1* across multiple species.**

|  |  | **NCBI annotation?**  (Yes or no for small variant missing exon 6) | **RNA-sequencing data confirming small variant detectable?**  **(eg. experiment accession)** |
| --- | --- | --- | --- |
| Apes | Humans  (*Homo sapiens*) | **Yes** | Yes - eg. bone marrow B cells (SRX4196499), peripheral B cells (SRX5358456, ERX1958871), liver (ERX1403333), CD8+ T cells (SRX5830986, SRX3527702), pDCs (SRX1921647), lung (SRX1853552, SRX4667994) , stem cells (SRX290739, SRX290726), skin (SRX4882388), intestine (ERX2322685), kidney (SRX4404718, SRX3051312), blood (SRX3823187, SRX3823188) |
|  | Chimpanzee  *(Pan troglodytes)* | **Yes** | Yes- eg. blood (SRX724891), lymphoblastoid cell line (SRX333019), liver (SRX081967), iPSC (SRX3333969, SRX290731), PBMC (SRX3291890), lung (SRX843158, SRX3849733) |
|  | Bonobo  (*Pan paniscus*) | **Yes** | Yes- eg. SRX290737 (iPSC), limited data available |
|  | Gorilla | **Yes** | Too few data available |
|  | Orangutan  (*Pongo abelii*) | **Yes** | No – iPSC only (1 project), too few data available |
|  | Gibbon (*Nomascus leucogenys*) | **Yes** | No data available |
| Old world monkeys | Rhesus macaque  (*Macaca mulatta*) | **Yes** | Yes - eg. bone marrow (SRX3130108, SRX584616), lung (SRX209572), liver (SRX209572) |
|  | Mangabey  (*Cercocebus atys*) | **Yes** | No- liver, spleen (limited samples)  Yes- eg. lung (SRX843258), reference mRNA (SRX270666), colon (SRX843254) |
|  | Baboon  (*Papio anubis*) | **Yes** | No- PBMC, lung, liver, LN, skin |
|  | Gelada baboon *(Theropithecus gelada)* | **Yes** | Too few data available |
|  | Black/golden snub-nosed monkey (*Rhinopithecus bieti/roxellana*) | No | No -lung, blood, liver, limited data available |
|  | Green monkey (*Chlorocebus sabaeus*) | **Yes** | Yes- eg. blood (SRX379187), COS7 cell line (SRX4891877) |
| New world monkeys | Marmoset  (*Callithrix jacchus*) | No | No- liver, lung, intestine, iPSC, reference mRNA |
|  | Night monkey  (*Aotus nancymaae*) | No | No- blood, bone marrow, liver, spleen, colon, LN, kidney –from 1 project |
| Tarsier | Philippine Tarsier  (*Carlito syrichta*) | No | No data available |
| Lemurs | Gray mouse lemur (*Microcebus murinus*) | No | No- lung, liver |
|  | Small-eared galago (*Otolemur garnettii*) | **Yes** | No -liver , too few data available |
| Other mammals | Mouse  (*Mus musculus*) | No | No- liver, lung, CD103+ or pulmonary DC, neutrophils, intestine, placenta |
|  | Rat  (*Rattus norvegicus*) | No | No- liver, lung, spleen) |
|  | Guinea pig  (*Cavia porcellus*) | **Yes** | No- liver, lung, kidney, cervix, skin |
|  | Dog  (*Canis lupus familiaris*) | No | No –liver, lung, kidney, T cells, placenta |
|  | Cow  (*Bos taurus*) | No | No- liver, lung, alveolar or bone marrow macrophages, skin, intestine |
