# Supplementary material for: Differential expression of interferon-lambda receptor 1 splice variants determines the magnitude of the antiviral response induced by interferon-lambda 3 in human immune cells
Source: PLoS Pathog. 2020 Apr 30;16(4):e1008515. doi: 10.1371/journal.ppat.1008515 (PMC7217487; doi:10.1371/journal.ppat.1008515)
Supplement: S4 Table — (DOCX) [file ppat.1008515.s011.docx]

**Table S4: List of SYBR RT-qPCR primer sequences**

| *Gene* | *SYBR Primer Sequence* |
| --- | --- |
| HPRT1 | F:5’-TGACACTGGCAAAACAATGCA-3’ |
|  | R:5’-GGTCCTTTTCACCAGCAAGCT-3’ |
| RPL13A | F:5’-CCTGGAGGAGAAGAGGAAAGAGA-3’ |
|  | R:5’-TTGAGGACCTCTGTGTATTTGTCAA-3’ |
| OAS1 | F:5’-CAAGCTCAAGAGCCTCATCC-3’ |
|  | R:5’-TGGGCTGTGTTGAAATGTGT-3’ |
| IFIT1 | F:5’-AGAAGCAGGCAATCACAGAAAA-3’ |
|  | R:5’-CTGAAACCGACCATAGTGGAAAT-3’ |
| ISG15 | F:5’-TGGTGAGGAATAACAAGGGC-3’ |
|  | R:5’-CAGATTCATGAACACGGTGC-3’ |
| IFI44 | F:5’-CCACCGAGATGTCAGAAAGAG-3’ |
|  | R:5’-TGGTACATGTGGCTTTGCTC-3’ |
| mIFNLR1 | F:5’-CACGGGCCCTGGACTTTTCT-3’  R:5’-CTGCAAGGTCCTTCTTCCATCTT-3’ |
| sIFNLR1 | F:5’-TGGAGGTCCCAGGACTTTTCTG-3’  R:5’-CTGCAAGGTCCTTCTTCCATCTT-3’ |
